# Lymph Nodes and Cancer Metastasis: New Perspectives on the Role of Intranodal Lymphatic Sinuses

**DOI:** 10.3390/ijms18010051

**Published:** 2016-12-28

**Authors:** Rui-Cheng Ji

**Affiliations:** Faculty of Welfare and Health Science, Oita University, Oita 870-1192, Japan; ji@oita-u.ac.jp; Tel./Fax: +81-097-554-7973

**Keywords:** lymph nodes, lymphatic endothelial cells, cancer metastasis, lymphangiogenesis, VEGF-A/-C/-D

## Abstract

The lymphatic system is essential for transporting interstitial fluid, soluble antigen, and immune cells from peripheral tissues to lymph nodes (LNs). Functional integrity of LNs is dependent on intact lymphatics and effective lymph drainage. Molecular mechanisms that facilitate interactions between tumor cells and lymphatic endothelial cells (LECs) during tumor progression still remain to be identified. The cellular and molecular structures of LNs are optimized to trigger a rapid and efficient immune response, and to participate in the process of tumor metastasis by stimulating lymphangiogenesis and establishing a premetastatic niche in LNs. Several molecules, e.g., S1P, CCR7-CCL19/CCL21, CXCL12/CXCR4, IL-7, IFN-γ, TGF-β, and integrin α4β1 play an important role in controlling the activity of LN stromal cells including LECs, fibroblastic reticular cells (FRCs) and follicular dendritic cells (DCs). The functional stromal cells are critical for reconstruction and remodeling of the LN that creates a unique microenvironment of tumor cells and LECs for cancer metastasis. LN metastasis is a major determinant for the prognosis of most human cancers and clinical management. Ongoing work to elucidate the function and molecular regulation of LN lymphatic sinuses will provide insight into cancer development mechanisms and improve therapeutic approaches for human malignancy.

## 1. Introduction

The lymphatic system plays an active role in modulating inflammation, autoimmune disease, and anti-tumor immune response. Antigens and antigen-presenting cells (APCs) including macrophages and dendritic cells (DCs) are transported by afferent lymphatics to the draining lymph nodes (LNs) [1,2]. The naïve T and B cells enter LNs via high endothelial venules (HEVs), which are specialized postcapillary venules found in secondary lymphoid organs for supporting high levels of lymphocyte extravasation from the blood [3]. The adhesion molecules, e.g., integrin, and chemokine receptors, e.g., chemokine (C–C motif) receptor 7 (CCR7) and chemokine (C–X–C motif) receptor 4 (CXCR4) regulate cell trafficking. Inside the LNs, naïve T and B cells home to distinct compartments, the T-cell areas and B-cell follicles, and then leave the LNs via efferent lymphatics [4]. Under pathological conditions, e.g., cancer and inflammation, LNs undergo dramatic remodeling including changes in immune cell trafficking and cellularity, and lymph flow [5]. Especially, tumor metastasis and inflammation share several features that result in alterations of the structure and function in LNs, including hyperplasia and lymphangiogenesis. Many human tumor cells appear to take advantage of the lymphatic system to promote metastasis to LNs and distant organs [6,7,8]. The tumor-associated lymphatics and draining LNs are key modulators of tumor cell migration and invasion, although the underlying molecular mechanism remains to be unraveled. Current opinion indicates that LN lymphangiogenesis plays an integral role in promoting the initial spread of malignant tumors. The procedure of sentinel LN mapping may be crucial for determining prognosis and treatment of cancer patients [9].

Lymphatic endothelial cells (LECs) are emerging as important players in modulating adaptive immune responses, presenting tumor antigen and inducing immune tolerance. In the dynamic interplay with tumor cells and immune cells, LEC function in controlling immune cell trafficking is important for immune surveillance during early stages of tumor development and may facilitate host protection against cancer. Otherwise, LEC ability to promote immunosuppression may induce immune escape of tumor cells, and thus accelerate tumor initiation and progression [10,11]. An emerging picture of LN metastasis is complex, and in many ways depending on how the tumor microenvironment affects cancer progression and LEC biology. Although the sensitivity of lymph node lymphatic endothelial cells (LN-LECs), the cells derived from intranodal lymphatic sinuses to the dynamic tumor microenvironment remains poorly understood, they should no longer be considered a passive bystander in tumor metastasis but rather an active player in many intercellular and intracellular processes [12,13]. This review aims to assess the remodeling and reconstruction, as well as immune involvement of LN lymphatic sinuses during tumor metastasis, with emphasizing the interaction between tumor cells and LN-LECs including lymphangiogenesis. LN-LECs represent attractive therapeutic targets to enhance tumor immunotherapy and to control tumor metastasis.

## 2. Lymphatic Sinuses and HEVs in LNs

LNs contain a lymphatic network for conducting pathogens, immune cells, metastatic tumor cells and lymph fluid. LN lymphatic sinuses consist of sinusoidal microstructures that begin at the peripheral afferent lymphatics, extending underneath the capsule and surrounding B-cell follicles, and then penetrating into the medullary region including T-cell areas [14]. Intranodal lymphatic sinuses and blood vessels serve distinct but complementary functions to maintain tissue homeostasis.

### 2.1. LN Stromal Cells

LNs are remarkably organized with T and B cell compartmentalization, APCs and stromal cells to provide optimal interactions of the immune system components with invading pathogens [1]. Stromal cells in LNs can be divided into several sub-populations by their expression of surface markers, which mainly include LECs, fibroblastic reticular cells (FRCs), and follicular DCs. The stromal cells are relatively stable and targetable, contributing to cancer progression through a complex crosstalk with cancer cells. LECs are nonhematopoietic stromal cells of lymphoid organs, lining peripheral lymphatics and guide migrating leukocytes toward the LN. LN-derived LECs are a second main population of stromal cells in LNs found in many compartments. Lymphatic sinuses lined by LECs occupy three distinct locations, the subcapsular, cortical, and medullary sinuses [15]. LN-LECs in the cortical sinuses regulate intranodal lymphocyte trafficking by collecting lymphocytes for further transit to medullary sinuses and eventual LN egress [16,17]. Lymphatic growth and development in LNs are positively regulated by B cells and macrophages [18]. However, the classification of stromal cell subsets still needs to be improved. The molecular phenotype and distribution pattern of LECs in human LNs have also been found to differ from that observed in murine LNs. Lymphatic vessel endothelial hyaluronan receptor (LYVE-1) is expressed by lymphatic sinuses throughout the murine LN. In contrast, LYVE-1 expression is only seen in LECs of the paracortical and medullary sinuses in the vast majority of human LNs, but negative in the subcapsular and trabecular sinuses [15,19]. Moreover, prospero-related homeobox 1 (Prox-1) is shown to be the only marker that uniquely identifies the LECs lining all the lymphatic sinuses in human LNs. APCs in the sinuses can be clearly distinguished from LECs by expression of CD169, and lack of expression of Prox-1 and STAB2 [20]. Lymphatic sinuses in LNs play a key role in trafficking of immune cells including pathogen-carrying CD11c^+^ DCs and CD169^+^ sinusoidal macrophages for adaptive immunity [21,22]. Moreover, the blood vessels run through the medullary region after entering LNs from the hilum, and are then distributed throughout the cortex. HEVs, specialized blood vessels in the paracortex, facilitate lymphocyte homing from systemic circulation to LNs [23]. The majority of HEVs are separate from the lymphatic sinuses, and thus the naïve T and B cells constantly circulate through secondary lymphoid organs via the postcapillary venules.

Within the LN, the primary follicles are composed of B cells with follicular DCs located in the cortex, whereas T cells and DCs are distributed in the paracortical area. Lymph enters the subcapsular sinus of the draining LN through afferent lymphatics and flows into the medullary sinus via a delicate network of conduits, formed by follicular DCs in the B cell zone and FRCs in the T cell zone, prior to leaving the LN via efferent lymphatics [19,24]. In particular, the podoplanin-expressing FRCs secrete extracellular matrix components including collagen, and form the mesenchymal backbone on which T cells, B cells and DCs migrate and interact [25]. Thus, FRCs and follicular DCs impose separation of lymphocytes into the characteristic paracortical T cell zone interrupted by discrete B cell follicles extending into the cortex. As a molecular sieve, the highly interconnected microtubules only allow small molecules and particles including antigens and inflammatory mediators to flow along the network [26]. The conduit system transports low-molecular-weight soluble antigens from the cortex to the paracortex and into the parenchyma of LNs, where they may contact the HEVs [19]. Lymph-borne chemokines likely adopt this route to regulate HEV function and rapidly change lymphocyte HEV transmigration in the paracortical T cell area [27].

As such, LNs represent the dynamic structure of high complexity that harbors different compartments with distinct microanatomical features, the lymphatic sinuses, T-cell zone and B-cell zone. Activated T cells begin emigrating from LNs through cortical sinuses and make their exit via efferent lymphatics [17]. Afferent lymph-derived T cells and DCs may use different chemokine receptor CCR7-dependent routes for entry into the LN and intranodal migration [28]. Developing T-cells interact with various types of stromal cells including blood vascular endothelial cells (BECs), but the interaction of T cells (mainly memory T cells) with LECs has not yet been investigated sufficiently. Moreover, B cells migrate along the FRC network to reach the lymph follicles, where the follicular DCs regulate B cell homeostasis, migration and survival [29]. The majority of LN macrophages generally reside in the marginal sinus and medullary cords [23].

Transport of lymph to the draining LN is achieved in such a way as to optimize the delivery of pathogenic signals, antigens, and immune cells to promote antigen capture by B cells and DCs, and facilitate T cell education [22]. Functional lymphatics are required for the interactions between APCs and antigen-specific lymphocytes to maintain the microarchitecture of LNs [14]. It is evident that nonhematopoietic stromal cells of secondary lymphoid organs form important scaffold and fluid transport structures, such as LN trabeculae, lymphatic sinuses, and conduits. Through the production of chemokines and cytokines, these cells generate a particular microenvironment for regulation of lymphocyte homeostasis and tumor progression and metastasis [30,31]. A better understanding of the cellular structures of LNs controlling immune cell trafficking is likely to lead to new insights into disease processes, and also improves the development of new immunomodulatory therapy.

### 2.2. Regulation of Cell Trafficking

LNs as an immunological filter are strategically situated throughout the mammalian body to trap particles or antigens from peripheral tissues [5]. LN stromal cells, e.g., FRCs and follicular DCs are reported to be important for trafficking of T and B cells [26]. Cellular trafficking in the periphery and LNs is mainly regulated by the CC chemokine ligand (CCL)-19 and CCL21, the chemokine (C-X-C motif) ligand (CXCL)-13, and the lysophospholipid sphingosine-1-phosphate (S1P) [1,32]. The lymphatics provide routes for DC and lymphocyte migration into and out of LNs, whereas LECs may control the process by expression of CCL19, CCL21, S1P, and adhesion molecules (Table 1).

#### 2.2.1. Chemokines

The compartmentalization of cells in LNs is orchestrated by lymphoid chemokines. The CCR7-CCL19/CCL21 axis has a key role in the formation of secondary lymphoid structures by recruiting immune cells under physiological conditions. CCL21 is produced by LECs, intranodal FRCs and HEVs, but CCL19 is secreted by FRCs within the LN [16,33,34]. CCL19 and CCL21, ligands for CCR-7 recruit and direct the distribution of CCR-7-expressing cells, mostly T cells and antigen-presenting DCs. APC movement to LNs is dependent on the constitutive chemokine receptor CCR-7, which is expressed by B cells, mature DCs and by several T-cell sub-populations including naïve, regulatory and central memory T cells [35]. CCR7-dependent migration of APCs, mostly DCs, to initial lymphatics, is primarily directed by a chemotactic gradient of CCL21 and interstitial flow [36]. Antigen-laden tissue-derived DCs enter LNs mainly via afferent lymphatics, and the afferent lymph-borne DCs essentially rely on CCR7 for their transition from the subcapsular sinus into the parenchyma of LNs [37]. DCs play crucial roles in maintaining HEV phenotype and regulating lymphocyte entry into the LN [38]. Distinct functional subsets of T cells enter LNs mainly through HEVs. After crossing HEVs, naïve T cells migrate to T cell zones in the LN paracortex, whereas naïve B cells expressing CXCR5 are induced to migrate into follicles in the cortex through follicular DC expression of CXCL13 [23].

#### 2.2.2. Integrins

LECs of afferent lymphatics express chemokines and adhesion molecules that facilitate the migration of DCs and T cells to LNs. Integrins expressed by HEVs also regulate leukocyte entry to LNs. Lymphatics and HEVs with expression of chemokines and adhesion molecules may serve as organizers of LN development [39]. Integrin α9 and its ligand, tenascin-C, colocalize on LECs lining cortical and medullary sinuses of draining LNs. Integrin α9 is critical for the development of lymphatic valves, which is further regulated by Notch signaling during valve morphogenesis [40,41]. Prox-1-induced LEC differentiation is mediated at least in part through integrin α9 [42]. In experimental models, blockade of integrin α9-mediated signaling has reduced lymphocyte egress from draining LNs [43], indicating that the colocalization and interaction between integrin α9 and tenascin-C affects the number of lymphocytes within lymphatic sinuses. The transit of T cells and APCs from tissue to draining LNs is also via intervention of the receptors, e.g., the common lymphatic endothelial and vascular endothelial receptor-1 (CLEVER-1) and the mannose receptor. CLEVER-1, a scavenger receptor expressed on lymphatics, is involved in trafficking of adoptively transferred T cells from the skin to LNs [44]. The mannose receptor, a C-type lectin carbohydrate binding protein, is primarily expressed on the surface of macrophages and DCs, and on LECs of both afferent and efferent lymphatics [45]. The absence of the mannose receptor may impair lymphocyte adhesion and trafficking [46].

#### 2.2.3. S1P

S1P, a lipid signaling molecule with its receptor controls the egress of B and T cells from LNs into the efferent lymphatics, and thus has emerged as a new player in tumor microenvironment and progression during the last decade. S1P modulates lymphocyte trafficking and immune responses, and regulates the maturation of lymphatic button and zipper-like junctions either directly or by lymphocyte-mediated mechanisms [47,48]. In the cortical sinus of LNs, LECs are identified as the cellular source of S1P [32]. Spinster 2, a S1P transporter, has shown to regulate LN and lymph S1P levels, and consequently influencing lymphocyte trafficking and lymphatic network organization. Impaired lymphocyte egress from LNs and aberrant intranodal lymphatic sinuses were found in spinster 2^−/−^ mice [49]. Pharmacological suppression of S1P production can inhibit the tumor-induced increase of LECs in the LNs [50]. LECs are capable of transducing signals from extracellular S1P that mediate lymphatic development and organization [49]. LN-LECs are also directly involved in dampening the immune response and tolerance induction [12]. Obviously, the role of LN-LECs in the regulation of immune responses goes beyond transporting antigen, lymphocytes and inflammatory mediators from peripheral tissues to LNs.

### 2.3. LN Remodeling and Reconstruction

Intact lymphatics are required for structural and functional maintenance of regional LNs, and for the trafficking of lymphocytes, DCs and macrophages. Abnormal lymphatic sinuses in LNs will greatly reduce the clearance of draining pathogens from peripheral tissues [51]. Activation of the endothelial cells comprising HEVs and lymphatic sinuses will quickly take place upon inflammatory insult, leading to angiogenesis and lymphangiogenesis and other changes [34,52]. The change of leukocytes and stromal cells during LN remodeling and reconstruction is a complicated interactive process in the regulation of several molecules and factors (Table 1). In the context of cancer progression, the lymphatic sinuses in draining LNs undergo dramatically morphological remodeling and functional changes. LN lymphangiogenesis and cellular contents may be easily influenced by growth factors, cytokines, and chemokines, e.g., vascular endothelial growth factor (VEGF)-A and CCL21 secreted by cancer cells and stromal cells in tumor microenvironment. The LN lymphangiogenesis may be actively involved in leukocyte migration and induction of immune responses during cancer progression [1].

#### 2.3.1. Interleukin-7 (IL-7)

Interleukin-7 (IL-7) is a cytokine crucial for LN organogenesis, and lymphocyte development and homeostasis. LECs and FRCs are a prominent source of IL-7 both in human and murine LNs. IL-7-producing LECs are in the LN medulla, but FRCs are in the T cell zone and cortical ridge of LNs [53]. FRCs produce IL-7 for promoting survival of naïve T cells [16]. During virus-induced LN remodeling, upregulated expression of IL-7 in LECs and FRCs was found to participate in the structural adaptation of the LN microenvironment. IL-7-producing stromal cells contribute to de novo formation of LYVE-1-positive lymphatic structures connecting the reconstructed LNs with the surrounding tissue [30]. Moreover, LECs also express the IL-7 receptor chains IL-7Rα. In several mouse models, IL-7 and IL-7Rα expression in stromal cells has indicated the changes in lymphatic morphology and lymph drainage. In IL-7Rα-deficient mice, the absence of most peripheral LNs may reflect the importance of IL-7 in LN development and maturation [54]. Near-infrared in vivo imaging performed in the lower limb of IL-7Rα^−/−^ mice has shown normal pumping activity of collecting vessels but defective fluid uptake into initial lymphatics [55]. During inflammatory processes, the structural reorganization of lymphatic sinuses impinges on lymphocyte dynamics in LNs [15]. Ectopic lymphoid-organ formation occurs through IL-7-mediated enhanced survival of lymphoid tissue inducer cells [56], suggesting that IL-7 overexpression may contribute to the adaptation of lymphoid organ structure during immune responses. In addition, during LN development, IL-7 is produced by vascular cell adhesion molecule 1 (VCAM-1)^+^ and integrin ligand intercellular adhesion molecule 1 (ICAM-1)^+^ mesenchymal cells, also known as stromal organizer cells [57]. Stromal cell-derived IL-7 promotes survival of lymphoid tissue inducer cells that initiate lymphotoxin-β receptor (LTβR)-dependent formation of the LN microenvironment [58].

#### 2.3.2. LTβR

LTβR is broadly expressed on both stromal cells and hematopoietic cells, including LECs, BECs, FRCs, follicular DCs, macrophages, and mast cells. LTβR signaling is implicated in the potential regulation of lymphatics, HEVs, and stromal cells [5,59]. The LN formation is a complex process controlled through triggering of LTβR on mesenchymal cells. LTβR expressed on stromal cells directly regulates the expression of adhesion molecules, chemokines, and cytokines relating to cell trafficking, LN homeostasis and remodeling [60]. Notably, LTβR induces cytokine expression and upregulates lymphangiogenic factors in LN anlagen. Activation of LTβR further induces VEGF-C production in stromal organizer cells, recruiting lymphatics toward the nascent LN [61]. The lymphoid tissue microenvironment is crucial for the maintenance of HEV characteristics during homeostasis and its relationship with lymphatics in the remodeling process after immunization [6,38,62]. Lymphatics and HEVs maintain homeostasis under steady state and undergo dramatic activation including lymphangiogenesis and angiogenesis during inflammation [5].

#### 2.3.3. C-Type Lectin-Like Receptor 2 (CLEC-2)

C-type lectin-like receptor 2 (CLEC-2), a receptor for podoplanin, is expressed by LN-LECs and FRCs, but not by blood vessels including HEVs in LNs [63]. CLEC-2 for binding *O*-glycosylated podoplanin has been found to be involved in the development and maintenance of LNs, and the migration of antigen-presenting DCs through lymphatics to T cell zones [64]. The physical elasticity of LNs is maintained in part by podoplanin signaling in stromal FRCs and its modulation by CLEC-2 expressed on DCs. The DCs that initiate immunity by presenting antigens to T cells also cause remodeling of LNs by delivering CLEC-2 to FRCs [65]. In LNs, FRCs form a collagen-based reticular network, which supports migratory DCs and T cells, and lymph drainage. CLEC-2 modulation of podoplanin signaling permits FRC network stretching and allows for the rapid LN expansion-driven by lymphocyte influx and proliferation, which represents a critical hallmark of adaptive immunity [65]. Disrupting podoplanin function alters the homeostasis and spacing of FRCs and T cells, which further results in an expanded reticular network and enhanced immunity [66]. Podoplanin is required to protect the barrier function of HEVs during lymphocyte trafficking, and local S1P release after podoplanin-CLEC-2-mediated platelet activation is critical for HEV integrity during immune responses. Mice with postnatal deletion of podoplanin have lost HEV integrity, showing spontaneous bleeding in mucosal LNs and draining peripheral LNs after immunization [63]. Therefore, the CLEC-2-podoplanin axis controls the contractility of FRCs and LN microarchitecture, as well as the functional integrity of HEVs.

**Figure 1:**
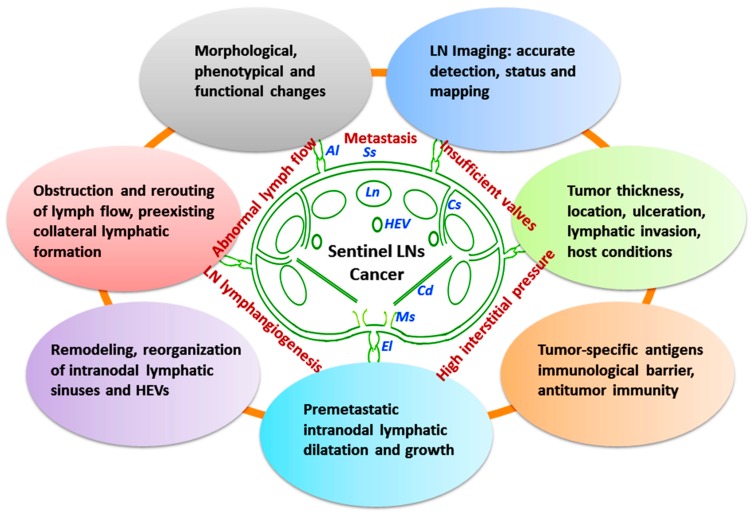
Lymph node (LN) remodeling and reconstruction. Sentinel LNs show obvious morphological and functional changes in tumor microenvironment. Al: afferent lymphatics; Ss: subcapsular sinuses; Cs: cortical sinuses; Ms: medullary sinuses; El: efferent lymphatics; Ln: lymph nodules; HEV: high endothelial venules; Cd: conduits.

The lymphatics provide an initial transport route for cancer cell dissemination, and regional LNs are regarded as the first site of metastasis in patients with tumor progression [31]. In preclinical studies, LN lymphangiogenesis occurs prior to the onset of metastasis and is accompanied by an increase in lymph flow to draining LNs, facilitating tumor cell entry into the lymphatics [6]. In premetastatic melanomas and lymphomas, intranodal lymphangiogenesis was found to be associated with greatly increased lymph flow, which actively drives tumor metastasis to draining LNs [77]. Extensive tumor invasion of the draining LN may obstruct or change normal lymph drainage pathway. Recently, non-invasively imaged changes of lymphatic function and drainage patterns have been reported by using near-infrared fluorescence (NIRF) imaging. In melanoma models, the dynamic NIRF imaging with sufficient spatial and temporal resolution and sensitivity has indicated significant reduction of lymphatic contractility and altered lymph drainage patterns [78]. The extent of B cell accumulation within the tumor-draining LNs may also predict the level of LN lymphangiogenesis and metastatic potential [79]. The immune cell accumulation and LN lymphangiogenesis phenotype may identify host anti-tumor responses for promoting tumor cell spread through lymphatics. The LN remodeling and reconstruction has thus provided a permissive microenvironment for cancer metastasis and immune responses (Figure 1).

## 3. LN-LECs and Cancer Immunity

The lymphatic system has the ability to quickly initiate protective immunity against foreign antigens upon stimulation, in which initial lymphatics collect interstitial fluid and DCs from the periphery and deliver them to the LNs for generation of adaptive immune responses, tissue immune surveillance and maintenance of peripheral tolerance. LNs are important centers for immune regulation where antigen-specific responses are directed against foreign and self-antigens from upstream peripheral tissues, leading to the generation of productive immunity against pathogens or the maintenance of immunological tolerance to self-antigens [80].

### 3.1. Immune Functions of LECs

**Figure 2:**
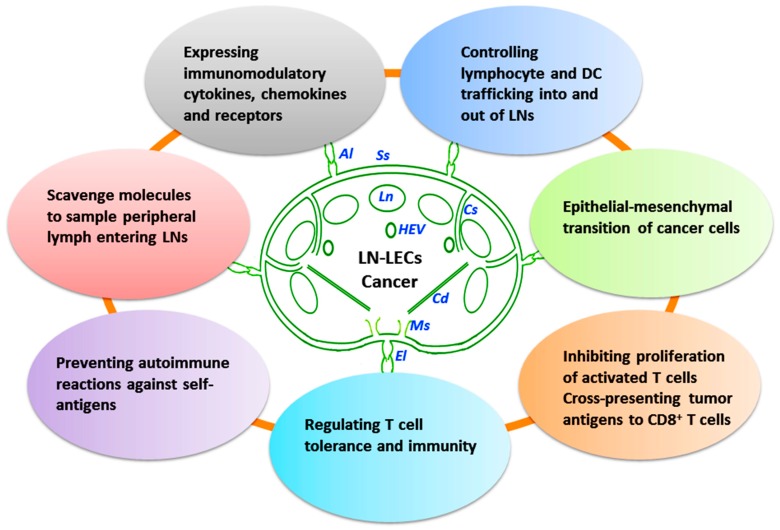
Involvement of LN-LECs in immune functions. LN-LECs in tumor microenvironment are actively involved in immunological responses. Al: afferent lymphatics; Ss: subcapsular sinuses; Cs: cortical sinuses; Ms: medullary sinuses; El: efferent lymphatics; Ln: lymph nodules; HEV: high endothelial venules; Cd: conduits.

Immune functions of LECs include cell transport from the periphery to LNs and constant sampling of peripheral antigens to APCs, e.g., DCs, macrophages, and B cells residing in the follicles. Actually, recent attention has been focused on the potential role of stromal cells, especially LECs in regulating immune responses and tolerance. LECs may contact with peripheral antigens and immune cells. These LECs can modulate DC function, present antigens to T cells on MHC class I and II molecules, and express immunomodulatory cytokines and receptors [17,81]. Increasing evidence has shown that LN-LECs are important in controlling the trafficking of DCs and lymphocytes, and in regulating T cell tolerance and immunity [12,82]. LN-LECs are involved in tumor-cell-epithelial mesenchymal transition, and interact with different immune cells within LNs and modulate their biological activity (Figure 2).

LECs are positioned in a strategic anatomical site where crucial interactions determining the fate of an immune response take place [10]. The LEC subpopulations with distinct molecular properties share a number of characteristics with professional APCs [82]. An emerging literature suggests an exciting paradigm shift for peripheral tolerance theory, in which naïve T cells trafficking through LNs are exposed to peripheral-tissue antigens (PTAs) specifically expressed by stromal cells [25]. In interfollicular regions and the medulla, LECs can impose antigen-specific deletional tolerance through direct presentation of PTAs to naïve CD8^+^ T cells [81]. In other words, LECs are integral for the maintenance of peripheral immune tolerance by suppressing autoreactive T cells in the LN. Under homeostatic conditions, LECs constitutively scavenge molecules to sample peripheral lymph entering LNs and actively cross-present exogenous antigens to CD8^+^ T cells. LECs help to maintain CD8^+^ T cell tolerance to exogenous antigens encountered in lymph for preventing autoimmune reactions against self-antigens after infection or injury [10]. LECs also actively enforce CD8^+^ T cell tolerance to PTAs through high-level expression of programmed cell death ligand-1 (PD-L1) and by lack of costimulatory molecules [82]. In cancers, LECs in the sentinel LN can cross-present tumor antigen MHC class I molecules leading to CD8^+^ T-cell deletion [2].

Lymph flow is a key mediator of lymphatic function, particularly with respect to DC recruitment and trafficking to LNs. Lymph flow delivers both APCs carrying antigen captured in the periphery, and soluble antigens, cytokines and pathogens for uptake by LN-resident DCs and macrophages. Lymph drainage also plays a crucial role in the organization and function of B cells resident in the draining LN, and in the regulation of humoral immunity and peripheral tolerance [83]. Thus, lymph flow through highly organized regions of the LN seems to act as a filter system that directs molecules or particles to specific regions for regulating immune responses to pathogens [80]. Increased lymph drainage attributable to blood vessel hyperpermeability occurs in tissue injury and acute inflammatory events, which may act as a preconditioning mechanism to affect LEC function in immune cell trafficking [71]. It is evident that the role of LECs in adaptive immune responses is far more extensive than previously realized in the biological and clinical implications. The immune function of LECs may explain why abnormal lymph drainage is correlated with autoimmunity and why tumor-associated lymphatics promote tumor progression and metastasis to distant organs [10]. LN-LECs acting as an active modulator of antitumor immunity may be a potential new target for cancer immunotherapy.

### 3.2. Immune-Related Factors of LN-LECs

The lymphatic system transports soluble antigens, chemokines, cytokines, and immune cells from peripheral tissues into draining LNs and back to the blood circulation after surveillance, thereby initiating adaptive immune responses. In this context, several factors are worthy of particular mention (Table 1).

#### 3.2.1. Nitric Oxide (NO) and Interferon-γ (IFN-γ)

Nitric oxide (NO) is a key effector molecule involved in immune regulation and defense. The endothelial nitric oxide synthase (eNOS) in LECs is physiologically required for robust lymphatic contraction, whereas inducible nitric oxide synthase (iNOS)-expressing bone marrow-derived CD11b^+^Gr-1^+^ cells suppress lymphatic contraction under inflammatory conditions [84]. NO is linked to the pathogenesis of various inflammatory and autoimmune diseases by affecting the development of T helper type 1 cells and regulatory T cells, and inhibiting T cell proliferation and cytokine expression in response to interferon-γ (IFN-γ) [85]. LECs have a tightly regulated iNOS-dependent mechanism to suppress CD8^+^ T-cell proliferation during inflammation. LECs and FRCs in the LN upregulate iNOS in the presence of activated T cells and secreted immunosuppressive NO [17]. The attenuated lymphatic function can prevent autoreactive T-cell activation and protect against autoimmune disease [84]. IFN-γ, a pleiotropic cytokine secreted by type 1 helper T cells, plays a major role in initiating cell-mediated adaptive immune responses and in modulating cell growth and differentiation. IFN-γ administration to athymic mice, significantly reduced lymphatic sinus density, and adoptive transfer of T cells from IFN-γ-deficient mice did not suppress the increase of lymphatic sinus density. T cells may negatively regulate LN lymphangiogenesis through IFN-γ that acts as a suppressor of lymphatic growth by downregulating the expression of LEC-specific genes [18]. Moreover, IFN-γ treatment may activate LN-LECs by specifically inducing surface expression of MHC class II molecules, but IFN-γ-stimulated LN-LECs fail to induce allogeneic CD4^+^ T-cell proliferation due to production of inhibitory molecules [67]. The combination of IFN-γ and tumor necrosis factor (TNF) produced by activated T cells has been demonstrated to drive iNOS expression and NO production by LECs. Although LECs come into direct contact with activated T cells at multiple locations in the LN, their influence on activated T cells is poorly understood [17].

#### 3.2.2. VEGF-A

VEGF-A induces LN lymphangiogenesis, hypertrophy, and HEV growth either directly via VEGFR-2 signaling or by upregulating lymphangiogenic factors, VEGF-C and VEGF-D [68,86]. Lymphangiogenic response and HEV expansion are involved in lymphocyte migration, especially DC mobilization leading to increased trafficking of APCs to draining LNs, thereby boosting immune responses [52,69]. VEGF-A may directly enhance immune responses, or in part through NF-κB activation and induction of cytokines and chemokines [87]. VEGF-A is also involved in immunosuppressive and immunomodulatory aspects via inhibiting T cell development and DC maturation, and in antigen clearance and inflammation resolution [68]. VEGF-C expression in tumor cells is correlated with metastasis and poor prognosis [70]. VEGF-C and LN lymphangiogenesis suppress antitumor immunity through local deletional tolerance, which in turn drives cancer progression and metastasis [70]. VEGF-C increases fluid drainage from primary tumors to the draining LN and antigen exposure to the adaptive immune system, in which VEGF-C-activated LECs might directly alter CD8^+^ T cell response [70]. However, the relative importance of antigen presentation by LECs in VEGF-C-driven tumor tolerance and immunity still remains to be explored.

#### 3.2.3. CCL21

The tumor-draining LNs are constantly bathed in tumor-associated chemokines and antigens, and APCs derived from tumor host. CCL21 expression by LECs and FRCs is modulated by VEGF-C and lymph flow in tumor microenvironment [71]. CCL21 normally functions as a lymphoid-homing chemokine, binding CCR7 on APCs and naïve T cells to direct them to the LN [35]. Increased lymph flow may act as an early inflammatory cue to enhance CCL21 expression by FRCs, thereby ensuring efficient immune cell trafficking, lymph sampling, and immune response induction. FRC sensitivity to lymph flow may help optimize the immune response before peripheral APCs reach the LN [72]. In the tumor stroma, CCL21 has a unique ability to attract and educate naïve T cells in the regulatory chemokine environment. The secretion of CCL21 was found to be correlated with invasiveness and immune tolerance in murine melanoma [73].

#### 3.2.4. D6 and Programmed Cell Death-1 (PD-1)

The chemokine-scavenging decoy receptor D6 expressed on LECs maintains lymphatic surfaces free of inflammatory CC-chemokines and prevents inappropriate leukocyte adherence [74]. In LNs, D6 immunoreactivity is expressed in the afferent lymphatics, and in subcapsular and medullary sinuses [88]. D6 plays an essential function in regulating lymph flow and cellular efflux from inflamed peripheral sites to draining LNs, and in contributing to the integration of innate and adaptive immune responses [75]. In inflamed contexts, D6 contributes to selective presentation of CCR7 ligands on LECs, and to regulation of LEC function to discriminate between mature and immature DCs [89]. D6 deficiency was found to be associated with lymphatic congestion and impaired movement of APCs from inflamed sites to LNs [75]. LN-LECs express multiple PTAs independent of the autoimmune regulator, which may induce programmed cell death-1 (PD-1)-mediated deletion of tyrosinase-specific CD8^+^ T cells [76,81]. The high-level expression of PTAs and PD-L1 is confined to LECs in the LN medullary sinus, and thus LN-resident LECs may serve as a quality control gatekeeper to determine the fate of emigrating CD8 T cells activated by other APCs [76]. LN lymphatic sinuses are involved in activating and directing immune cells, in sensing and responding to subtle changes in the LN microenvironment, and in modulating the balance between immunity and tolerance.

## 4. Lymphatics and Cancer Metastasis

**Figure 3:**
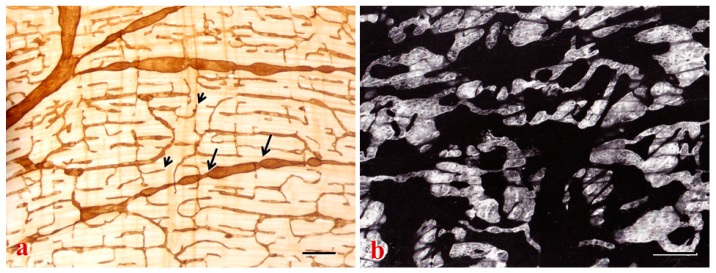

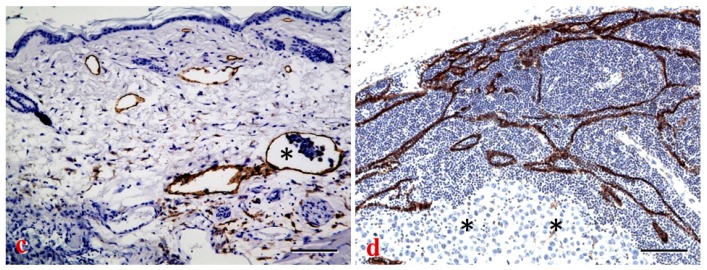
Lymphatics in normal and tumor tissues. (**a**,**b**) Lymphatic networks with 5′-Nase staining are featured by blind ends (arrowheads) and valves (arrows) in the intermuscular layer of the jejunum (**a**) and the pleural membrane (**b**), backscattered electron imaging in scanning electron microscopy) of monkeys; (**c**,**d**) In the mouse melanoma model, lymphatic vessel endothelial hyaluronan receptor (LYVE-1) staining shows increased lymphatic vessels in the skin (**c**), and increased subcapsular and cortical lymphatic sinuses in the lymph node (**d**). The metastatic cells invade lymphatic vessels in the subdermal tissue ((**c**), asterisk) and aggregate in the LN parenchyma ((**d**), asterisks). Bars: (**a**) 500 μm; (**b**) 150 μm; (**c**,**d**) 100 μm.

**Figure 4:**
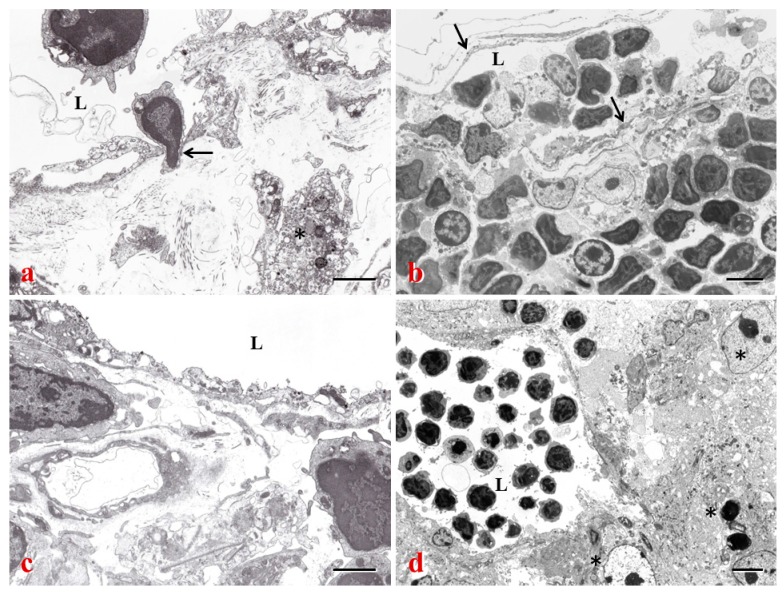
Peripheral lymphatic vessels (**a**) and intranodal lymphatic sinuses (**b**–**d**) in mouse tissues. (**a**) In the non-obese diabetic (NOD) pancreas, a lymphocyte is penetrating the lymphatic vessel stained with 5′-Nase cerium (arrow). The asterisk indicates a macrophage; (**b**) In the LN of Bagg albino/c (BALB/c) mice, the subcapsular sinus is filled with lymphocytes and DCs. The arrows indicate endothelial layers of the lymphatic sinus; (**c**) The medullary sinus of NOD mice is decorated with 5′-Nase cerium particles, and metabolic products of inflamed cells are seen beneath the endothelial layer; (**d**) The cortical sinus filled with lymphocytes is surrounded by metastatic melanoma cells (asterisks). L: lymphatic vessels or sinuses. Bars: (**a**,**c**) 2 μm; (**b**,**d**) 5 μm.

The lymphatic vessels lined by a single layer of endothelial cells, possess typical valves and intercellular junctions, and play a crucial role in cancer progression and metastasis (Figure 1, Figure 2, Figure 3 and Figure 4). In this context, the oncological involvement of LN-LECs is correlated with lymphangiogenesis, and immune suppression and tolerance. Tumor-associated intranodal lymphangiogenesis promotes the metastatic process by increasing the number of routes to LNs [6,79]. The LN status of solid tumors is one of the key parameters for determining the stage of tumor progression and treatment options [9].

### 4.1. Tumor-Involved Sentinel LN Status

The primary tumor greatly influences the microenvironment of sentinel LNs to promote tumor cell proliferation and subsequent metastases. The sentinel LN in solid tumors acts as an important gateway for malignant cells to spread to distal organs. It is the site where immunoreactive lymphocytes initially encounter tumor-specific antigens and develop antitumor immunity, and thus constitutes a primary immunological barrier against tumor metastasis. Sentinel LN metastasis usually indicates the first step in the spreading of cancer from the primary site of many malignancies. The establishment of metastasis to the sentinel LN might not be simply reflected by lymph dynamics and cancer cell transport. The sentinel LN metastasis is a series of complex interrelated steps and processes [9]. A variety of bioactive molecules (cytokines, chemokines and growth factors) are produced in the local tumor microenvironment by different cells, including LECs and tumor cells themselves. A correlation between VEGF-A/-C expression by the primary tumor and metastasis to the sentinel LN has been reported in several experimental models [68,90]. As compared to nonsentinel LNs in the same basin, the sentinel LN undergoes morphological, phenotypical and functional changes induced by the primary tumor [91]. The sentinel LN may have critical roles in the development of local immunity that could reject and eradicate metastatic cancer cells.

#### 4.1.1. Structural and Functional Remodeling of Sentinel LNs

The premetastatic remodeling or reorganization of lymphatic sinuses and HEVs in sentinel LNs is a common phenomenon independent to the metastatic ability of cancer cells in animal tumor models, and even in human tumors. Tumor-induced sentinel LN lymphangiogenesis occurs before metastasis, suggesting that the LN changes may precede arrival of cancer cells [92]. In cases of tumor-free LNs, the increased lymphatic network of sentinel LNs is a very early premetastatic sign and may provide a new prognostic indicator and target for aggressive diseases [93]. In the popliteal sentinel LN of mice bearing human nasopharyngeal carcinoma, intranodal lymphatic sinuses and HEVs are clearly dilated before metastasis. The enlargement of lymphatic sinuses containing very few cellular structures in sentinel LNs is correlated with the primary tumor weight, indicating that the tumor lymph drainage may induce a persistent alteration of lymphatic sinuses [94]. Tumor-induced immune modulation of sentinel LNs was also found to be related to tumor cell survival and metastasis development, and immune suppression was present before metastasis in sentinel LNs of melanoma patients [95]. Decreased immune response in the positive sentinel LN from melanoma patients is featured by decreased CD8^+^ effector T cells and stimulatory CD11c^+^CD86^+^ DCs, and increased forkhead box P3^+^ (Foxp3^+^) regulatory T cells. The density of FOXP3^+^ lymphocytes has been shown to be associated with progression and survival in patients with positive sentinel LN status [95,96]. Moreover, the metastatic tumor cells are a major source of the lymphangiogenic factors, VEGF-C and VEGF-A, and their expression in the metastasis frequently exceeds that of the corresponding primary tumors and mononuclear cells in the adjacent residual LN parenchyma or in naïve LNs [97,98]. A recent study has shown that the acidic microenvironment in tumors promotes proliferation and functional changes in LECs through activation of the transient receptor potential vanilloid subtype 1 (TRPV1), which may in turn lead to up-regulation of lymphatic metastasis [99].

Abnormal lymph flow, increased interstitial pressure, and insufficient function of lymphatic valves in tumor microenvironment may greatly influence cancer progression and metastasis. In solid tumors, the formation of increased interstitial pressure is multifactorial, e.g., increased vascular permeability and solid stress generated by proliferating tumor cells. Increased interstitial pressure may induce the development of hypoxia within tumor bulk, leading to reduced efficacy of therapeutic drugs and radiotherapy [13,100]. In different tumor types, the density and abnormality of peritumoral and intratumoral lymphatics vary and correlate with the incidence of LN metastasis and poor prognosis [101]. Abnormal lymphatics, especially lack of functional intratumoral lymphatics in tumor bulk may lead to further accumulation of fluid and proteins in the interstitium [102]. In many solid cancers, the structural properties of initial lymphatics, e.g., absent or discontinuous basement membrane, open or overlapping intercellular junctions, and tumor antigenicity detection by host immune cells, may account for why LN metastasis precedes blood metastasis [6,62]. Indeed, several tumor types, e.g., melanoma, breast cancer, and squamous cell carcinoma (SCC) are prone to metastasize to regional LNs through tumor-associated lymphatics. In a melanoma footpad model, the tumor-draining lymphatics were found to be dilated but remained functional, despite lower pulse rates. Lymphatic dysfunction induced by metastatic tumor spread to sentinel LNs at a certain size, may result in obstruction or rerouting of lymph flow away from the metastatic LN to alternately different LNs [103]. This rerouting might have been due to de novo formation of lymphatics or preexisting collateral lymphatics for allowing the compensation access. Tumor-associated LN remodeling and newly formed intranodal lymphatic route have indicated that the growth of metastases may accelerate tumor spread to alternate LNs [104]. HEVs, moreover, undergo a phenotypic change and functional shift, acting as a major gateway for lymphocyte infiltration into tumor tissues. In the study of human SCC of the tongue, significantly high density of abnormal HEVs was found in patients with established LN metastasis. The alteration of HEVs with increased endothelial proliferation may be correlated with tumor progression and clinical outcome [62]. The enlarged and remodeled HEVs can integrate into the metastatic tumor vasculatures with further differentiation, characterized by the gradual loss of their specific marker MECA-79 from the tumor margin to the central part of the metastatic tumor nest [94]. The LNs undergo remodeling with changes in complex kinetics of cell components and gene expressions in the endothelial cells of HEVs and intranodal lymphatic sinuses. However, the potential remodeling and functionality of tumor-draining LNs have remained to be determined.

#### 4.1.2. Detection and Mapping of Sentinel LNs

Metastatic spread to regional LNs is one of the earliest events of tumor cell dissemination and presents a most significant prognostic indicator for determining management and predicting survival of cancer patients [105]. In recent years, the study of LN metastasis in experimental models and human patients has provided valuable information concerning lymphatic biology, tumor cell migration, and sentinel LN mapping [106,107]. The sentinel LN involvement is consistently related with increasing tumor volume, but possibly with host conditions, tumor location, ulceration, increasing mitotic rate, and lymphatic invasion. The sentinel LN mapping and biopsy was first applied to melanoma, and subsequently extended to breast cancer and others [9]. Lymphatic imaging techniques are based on visualization of lymphatics and LNs in simple and minimal invasive procedures with less side-effects and repetitive application [107,108]. The therapeutic decision-making for cancer patients usually depends on accurate detection and mapping of sentinel LNs. The status of metastatic sentinel LNs serves as an indicator of tumor staging, patient management, and prognosis. New optimized techniques, e.g., nanoparticle-enhanced magnetic resonance imaging (MRI), quantum-dot-based fluorescence imaging, and near-infrared (NIR) imaging have been performed for functional assessment of lymph rerouting in tumor tissues after sentinel LN metastasis, and for dual-modality mapping guided photothermal therapy in regional LN metastasis of melanoma, breast and pancreatic cancers [103,106]. Furthermore, molecularly specific imaging has been used for targeting cancer-positive LNs and malignant cells with intravenous administration of radiolabeled and fluorescently labeled antibodies [109]. Intraoperative fluorescence imaging modalities were also reported by using light-absorption correction, macrophage-specific fluorescent probe (MFP) and albumin labeling for quantitative nodal staging. The metastasized LNs with significantly reduced macrophages lack uptake of MFP and generally show a low fluorescence signal [107,110]. Undoubtedly, the combined use of traditional non-invasive and novel quantitative imaging approaches may provide more rapid, accurate and detailed information for tumor-draining LN metastasis and obviate routine LN dissection for cancer staging.

### 4.2. LEC-Tumor Cell Interface and Prometastatic Factors

It is not totally unclear why the tumor cells ”choose” the lymphatics for migration, and how the tumor cells interact with LECs and get access into the initial lymphatics in the intratumoral and peritumoral tissues. The expansion of intranodal lymphatic sinuses, LN lymphangiogenesis, and increased lymph drainage may implicate that tumor tissues programmed for LN metastasis have exerted a wide range of effects on premetastatic adaptation of draining LNs [77,80]. The pro-tumorigenic changes, like increased tumor cell proliferation and abnormal LN morphology may be a response to diverse stimuli ranging from chemokines, cytokines and growth factors (Table 2). In particularly, the morphological changes of lymphatic sinuses in LNs might be a promising strategy for the early detection of cancer metastasis. In human ductal mammary carcinomas, LECs react specifically with tumor-derived products of lipoxygenases that may serve as a mediator of tumor cell invasion into lymphatics and metastatic spreading from the sentinel to postsentinel LNs. The process involves premetastatic niche in axillary LNs, invasion of tumor cells into the lymphatics, and eventually intranodal tumor cell arrest and extravasation [98]. The image analysis in a mouse tumor model has shown that fluorescently labeled breast cancer cells enter lymphatics initially as single cells and then occur as tumor cell clusters within the vessel [105]. Moreover, bulk invasion of metastatic cells in mammary carcinomas was detected by in vivo microscopy, showing that xenografted tumor cells spontaneously form mobile cohesive groups and preferentially invade into lymphatics. It suggests that single cell motility is essential for blood-borne metastasis while cohesive invasion is capable of lymphatic spread [111]. Currently, there still lacks sufficient evidence to assess the role of cellular and molecular events on tumor progression and LN metastasis.

#### 4.2.1. Transforming Growth Factor-β (TGF-β), an Inducer of Epithelial-Mesenchymal Transition (EMT)

Tumor cell heterogeneity is of importance in determining LN metastasis. Subgroups of cancer cells in a tumor have great differences in genetic alteration patterns, and in epigenetic and metabolic regulation. The microenvironment changes may confer distinct growth advantages, and diverse phenotypes with differing migration and metastatic capability may evolve in a tumor mass over time [112,113]. Certain malignant cells within a heterogeneous tumor preemptively penetrate into the slender lymphatic wall and invade the surrounding tissue, and subsequently metastasize to distal sites. The process of cancer cell invasion and metastasis has been implicated to be associated with epithelial-mesenchymal transition (EMT) activation, involving transdifferentiation of epithelial cells into mesenchymal-like cells with migratory and stem cell properties [114]. Tumor invasion into lymphatics may share some features with the processes similar to EMT in embryo implantation and embryogenesis, such as disruption of cell–cell adherens junctions and enhanced migratory capacity [115,116]. Transforming growth factor-β (TGF-β), a multifunctional cytokine, is known to play an important role in tumor progression and metastasis by promoting tumor angiogenesis and lymphangiogenesis as well as extracellular matrix formation [13,117]. TGF-β is also a potent inducer of EMT during development and in cancer, and may be essential in the selection of cohesive or single-cell migration pattern by metastatic cells [111,118]. The cellular source of TGF-β is attributed to immune cells, like macrophages and regulatory T cells in tumor microenvironment [119]. TGF-β1 can promote CCR7/CCL21-mediated crosstalk between tumor cells and LECs, and increase CCR7 expression in EMT cells through p38 mitogen-activated protein kinase (MAPK)-mediated activation of the JunB transcription factor. The mammary tumor cells undergoing TGF-β-induced EMT have been found to become activated for targeted migration through the lymphatics similar to DCs during inflammation, and thus acquire the capacity to detach and migrate away from the primary tumor. The migration process of EMT cells into lymphatics is dependent on the induction of CCR7, which stimulates the cells to sense and migrate toward CCL21 produced by LECs [120]. TGF-β signaling can also be enhanced by the sine oculis homeobox homolog 1 (SIX-1) expressed in tumor cells [121]. The upregulation of SIX-1 in primary tumor may increase peritumoral lymphatic density, promote lymphangiogenesis and LN metastasis in cervical cancers by increasing the expression of VEGF-C [122,123]. TGF-β triggers a complex network of signaling cascades including its crosstalk with integrins. In a mouse model of non-small-cell lung cancer, combined targeting of TGF-β and integrin-β3 has significantly reduced the incidence of LN metastasis [124]. In addition, TGF-β1 might exert dual effects on promotion and inhibition of lymphangiogenesis, by upregulating VEGF-C expression, and by downregulating VEGFR-3 and suppressing LEC properties [125,126]. These signals might thus be a potential therapeutic target for preventing LN tumor metastasis.

#### 4.2.2. The Role of Chemokines in Trafficking of Tumor Cells to LNs

Chemokines are actively involved in not only maturation and migration of immune cells, but the trafficking of tumor cells to LNs. Chemokine receptors in tumor cells have been shown to facilitate organ-specific metastasis in experimental tumor models and human cancers [127,128]. Tumor cells are guided into the lymphatics by chemokine gradients, in which CCL19 and CCL21 are key players in metastatic dissemination of malignant cells via the lymphatic system. CCL21 is constitutively expressed by the lymphatics and its receptor CCR7 is expressed by melanoma and breast cancer cells [129]. CCR7 expression on tumor cells promotes lymphatic spread in several malignant tumors, although a comprehensive characterization of the CCR7-CCL19/CCL21 axis in LN metastasis still remains to be determined. Experimental evidence has indicated that the signaling axis is primarily responsible for LN metastasis formation by recruiting tumor cells to the T cell zone of LNs [130,131]. Lymphatic invasion by CCR7 expressing pancreatic ductal adenocarcinoma (PDAC) cells is additionally enhanced by upregulation of CCL21 in tumor-associated lymphatics. Lack of CCR7 impairs the metastatic potential of PDAC [132]. In human breast cancers and esophageal SCC, causality analysis between CCR7 expression and LN metastasis has shown that CCR7 expression is significantly correlated with poor outcomes in patients [127]. Moreover, LECs lining afferent lymphatics and intranodal lymphatic sinuses represent a barrier for entry of tumor cells into the LNs, which is controlled by CCL1-CCR8. In human and mouse tissues, CCL1 secreted by LECs lining subcapsular sinuses was found to mediate the LN entry of CCR8-expressing melanoma cells from afferent lymphatics. CCL1 production and tumor cell migration to LECs are also promoted by proinflammatory mediators TNF, IL-1β and lipopolysaccharide. CCR8 inhibition results in tumor cell arrest in collecting lymphatics at the junction with the LN subcapsular sinus [133]. Recently, CXCL12 (stromal cell-derived factor-1, SDF-1)/CXCR4 system has been reported to be involved the establishment of LN metastasis in different cancers, e.g., breast cancer and SCC, and ovarian cancer [128,134]. The LECs express CXCL12, binding to CXCR4 receptor on malignant cells lead to chemoattraction. CXCR4 signaling may regulate metastasis of chemoresistant melanoma cells by a lymphatic premetastatic niche [135]. In preclinical mouse models, antagonists or neutralizing antibodies of CXCL12/CXCR4 axis as potential therapeutic reagents have been shown to reduce LN metastasis of breast cancer cells and melanoma cells [135].

#### 4.2.3. Involvement of Macrophages and Adhesion Molecules in Cancer Progression and Metastasis

Macrophages promote cancer initiation and progression by releasing cytokines. Alteration of macrophage phenotype and function has a profound effect on inflammatory tumors, in close association with macrophage-induced lymphangiogenesis [136,137,138]. The macrophage recruitment to the tumor microenvironment may activate the transcriptional factors (STAT3, HIF-lα, AP-1 and NF-κB) in cancer cells, which further enhance the production of several inflammatory mediators, cytokines and enzymes to accelerate LN metastasis [13,139]. Tumor-derived IL-1 has been reported to promote lymphangiogenesis and LN metastasis through M2-type macrophages. The IL-1α-driven inflammatory activation of lymphangiogenesis seems to provide a tumor microenvironment favorable for LN metastasis of lung cancer cells through crosstalk with macrophages [140]. In addition, adhesion molecules, e.g., integrin α9, ICAM-1 and VCAM-1, are essential for lymphatic development and function, mediating vessel stability, permeability, valve formation and migration of leukocytes to draining LNs [35,40]. LEC-expressing integrin α4β1 facilitates metastasis by promoting direct adhesion of tumor cells to expanded LECs in LNs, in part serving as a receptor to capture VCAM-1^+^ metastatic lung and breast tumor cells [141]. LECs may be involved in inhibiting DC maturation and function. Skin-derived LECs interact with DCs via ICAM-1 and diminish their ability to stimulate T cell responses [142]. Activated leukocyte cell adhesion molecule (ALCAM, CD166) is expressed by LECs and leukocytes in LNs, playing a crucial role in stabilizing LEC-LEC interactions, and in the organization and function of lymphatic networks and LN homeostasis. Abnormalities of the lymphatic network and DC migration may account for the reduced LN cellularity observed in ALCAM^−/−^ mice [143]. The upregulation of ALCAM in tumor tissues is closely correlated with cancer cell progression and metastasis [144].

#### 4.2.4. Other Molecular Mediators of LEC-Tumor Cell Interface

Tumor cells express multiple Toll-like receptors (TLRs) indicating their role in tumor progression and manipulation of host immune responses [145]. TLR deficiency is involved in decreased lymphangiogenesis and macrophage infiltration, decreased interstitial and lymphatic transport, and abnormal lymphatic architecture [146]. Stimulation of LECs by using specific TLR agonists may lead to distinct cytokine and chemokine secretion profiles that reflect the specialized functional requirements of LECs. Aberrant lymphatic growth and expansion help to provide a permissive immune niche for promoting tumor aggressiveness and LN metastasis [147]. The experimental study has indicated that microRNAs bind to TLRs to induce prometastatic inflammatory response that ultimately may promote tumor growth and metastasis [148]. In patients with LN invasion and resectable gastric cancer, a high TLR3 expression defines a population with even worse prognosis and survival [149]. Recently, an unbiased approach based on molecular mimicry has been developed to identify specific receptors that mediate LEC-melanoma cell interactions and metastasis. Validation experiments showed that the phosphatase 2 regulatory subunit A, α-isoform (PPP2R1A) is expressed on the cell surfaces of both melanoma and LECs in vitro as well as independent melanoma patient samples. PPP2R1A-PPP2R1A homodimers occurred at the cellular level to mediate cell–cell interactions at the lymphatic-tumor interface, indicating that PPP2R1A is a new biomarker for melanoma metastasis [150]. New molecular methods will allow investigators to label and track single tumor cells migrating in the lymph flow especially after they have reached draining sentinel LNs. In this context, a better understanding of the active interaction between LECs and cancer cells during tumor progression will guide the development of more effective approaches for successfully controlling and treating cancer.

### 4.3. LN Lymphangiogenesis and Metastasis

During the past decade, advances have dramatically increased the knowledge of the mechanisms of lymphangiogenesis in LNs, including the roles of lymphangiogenic factors and signaling cascades (Table 2). Metastatic spread is enhanced by increased lymphangiogenesis in and around the primary tumor. Tumor lymphangiogenesis is a multiple-factorial process attributed to the interactions between endothelial cells, tumor cells, and other components in tumor microenvironment. Lymphangiogenesis is also an important physiological response to inflammatory insult. Immune cells including macrophages, DCs, B cells and T cells have been reported to orchestrate lymphangiogenesis in inflamed peripheral sites and LNs [21,69]. Elucidating the interaction between lymphangiogenesis and LN pathobiology will bring us new insights into mechanisms of sentinel LN metastasis.

#### 4.3.1. LN Lymphangiogenesis and “Lymphovascular niche”

Tumor-induced lymphangiogenesis provides an expanded lymphatic network that increases the probability for tumor cells to enter into initial lymphatics in response to LEC-derived chemokines [11], and enhances molecular and cell delivery to the draining LN. The formation of new lymphatic sprouts surrounding tumor cells, and tumor cell transmigration through intercellular junctions of endothelial cell monolayers may explain the entry of tumor cells into initial lymphatics [104]. Cancer cells can actively invade lymphangiogenic vessels inside the LNs by inducing the formation of circular defects in the endothelial layer [98]. In hybridoma-induced tumor models, the tumor cells with their metabolic products were frequently found to adhere to or penetrate newly formed lymphatics in various patterns, especially near the intercellular junctions where dense 5’-Nase-cerium precipitates extend into the typical interdigitating and overlapping junctions [102]. Tumor-induced lymphangiogenesis in the vicinity of primary tumors increases the likelihood that invasive tumor cells will enter the lymphatics and traffic to regional LNs and beyond. In more detail, intratumoral lymphatics are usually physically collapsed and non-functional, cancer cells predominately disseminate by invading and utilizing peritumoral lymphatics. Lymphangiogenesis in the peritumoral tissues has been found to increase sentinel LN metastasis and postsentinel tumor spreading in several experimental models and clinical studies [102,151]. However, lymphatics in the tumor periphery have exhibited slow pulse rate and abnormal draining patterns as a result of dysfunctional valves in the dilated vessels [152]. Possibly, chronic exposure to factors draining from tumor tissues, e.g., NO or inflammatory mediators, may have direct effects on the dilation and contractility of collecting lymphatics [84].

Molecular factors secreted by tumor cells may alter the local microenvironment to promote invasion and metastasis. Tumor-draining sentinel LNs undergo enhanced lymphangiogenesis even before metastasis, acting as a permissive “lymphovascular niche” to ensure long-term survival, growth and colonization of metastatic cells in the secondary site [92]. Lymphatic involvement by metastatic tumor cells and LN lymphangiogenesis may further promote tolerance from host immunity. The draining LN is bathed in tumor interstitial fluid containing inflammatory mediators and tumor-secreted antigens, cytokines and growth factors. The tumor-draining LN is more immunologically tolerant of the invading cancer cells compared to the LN that does not drain the tumor [80], and it may partially explain the reason why sentinel LN lymphangiogenesis promotes metastasis.

#### 4.3.2. Prolymphangiogenic Factors and Metastasis

VEGF-C is one of the crucial factors in promoting lymphangiogenesis and lymphatic metastasis in several kinds of cancers. Primary tumors via secretion of VEGF-C induce lymphatic hyperplasia of the sentinel LN and thereby contribute to cancer progression. VEGF-C-induced sentinel LN lymphangiogenesis may promote the entry of tumor cells into afferent lymphatics and improve tumor cell survival inside the LN. In the rat breast cancer model, tumor-derived VEGF-C draining to regional LNs promotes the outgrowth of LN metastasis and induces lung metastasis independently of its effects on LN metastasis [153]. In the B16 murine melanoma model, VEGF-C-overexpression in cancer cells markedly increases lymph flow from the primary tumors to the draining LNs and subsequently increases the incidence of metastasis [154]. In addition, VEGF-C/phosphatidylinositol 3-kinase α (PI3Kα)-driven remodeling of LNs promotes premetastatic niche formation by activating integrin α4β1 [141], suggesting that VEGF-C may stimulate LN lymphangiogenesis by promoting PI3Kα-mediated integrin α4β1 activation. In the process of tumor-induced lymphangiogenesis, abnormal, nonfunctioning or immature lymphatic formation around tumor periphery may allow retrograde lymph drainage, but this concept has not yet been adequately demonstrated.

The elastin microfibril interface-located protein 1 (EMILIN-1), an extracellular matrix glycoprotein, is diffusely expressed in several tissues, exerting a suppressive or protective role in lymphatic formation, and tumor growth and metastatic spread to LNs. EMILIN-1 structural integrity represents a regulator of fundamental processes, e.g., tumor phenotype and dormancy, and premetastatic niche formation [155]. EMILIN-1 deficiency causes lymphatic hyperplasia and structural anomalies, facilitating lymphangiogenesis-related tumor cell dissemination to LNs [155]. Apelin, a bioactive peptide known as the ligand of the G protein-coupled receptor APJ, has been reported to regulate lymphangiogenesis and attenuate inflammation by promoting LEC function [172]. Apelin overexpression in malignant cells is associated with accelerated in vivo tumor growth, and increased intratumoral lymphangiogenesis and LN metastasis [156]. In tumor-bearing animals, administration of erythropoietin can stimulate both intranodal lymphangiogenesis and LN metastasis by increased migration, capillary-like tube formation, and dose- and time-dependent proliferation of human LECs, and can also increase VEGF-C expression in LN-derived CD11b^+^ macrophages [157].

SDF-1-expressing DCs have significant effects on the accumulation of CXCR4-expressing Lewis lung carcinoma cells, and on the formation of the regional LN premetastatic niche [158]. In the experiment, the cyclooxygenase-2 (COX-2)/prostaglandin E2-EP3-signaling dependent VEGF-C/-D production in macrophages and DCs has been suggested to induce lymphangiogenesis in the efferent side of subcapsular regions [158]. Inhibitors of prostaglandin E2, together with SDF-1 receptor antagonists and EP3 antagonists, may be promising agents for the suppression of premetastatic niche formation and LN metastasis. Ectopic expression of δ-catenin, an adherens junction protein, in LECs may increase cell motility and lymphatic network formation in vitro and lymphangiogenesis in vivo through modulation of small Rho GTPase activation. Knockdown of δ-catenin results in a significant reduction of lymphangiogenesis and tumor metastases in mouse models [173]. In addition, a recent experiment has shown that the lymphatic endothelial antibody of the murine chloride channel calcium-activated 1 (mCLCA1) protein induces proliferation and growth of intranodal lymphatic sinuses [174]. Undoubtedly, tumor lymphangiogenesis is closely related with cancer cell spread via the intranodal lymphatic channels. The sentinel LN may provide a permissive microenvironment for tumor progression and metastasis. However, the involvement of tumor-associated lymphangiogenesis in LN premetastatic niche formation and immune modulation during cancer progression remains to be clarified.

## 5. The Role of Intranodal Lymphatic Sinuses in Cancer Therapy

Structural and functional changes of the draining LNs are involved in several human diseases like lymphedema, inflammation and tumor metastasis. In tumor microenvironment, tumor cells, inflammatory cells and LECs play a crucial role in neoplastic transformation, cancer invasion and metastasis, host immunity, and therapeutic resistance through the release of extracellular signals e.g., cytokines, chemokines and growth factors [138]. Many cellular and molecular components of the microenvironment are emerging as potential targets for therapeutic strategies.

### 5.1. Inhibitors of Lymphatic Metastasis

The metastatic process involves a complex series of dynamic and reciprocal interactions between the tumor and the host, and thus the patient's immune system is intimately involved in these interactions. Recent advances in the study of LN biology are beginning to yield significant new insights into the mechanism of cancer metastasis and therapeutic implications, in which VEGF-C/VEGFR-3, Notch, TGF-β/BMP, MAPK, PI3K/Akt/mTOR signaling pathways have been described in the control of lymphatic growth and remodeling [31,141,160,175]. Therefore, targeting these common signaling pathways may be an attractive strategy for cancer therapy including immunotherapy (Table 2). Any means that eradicates cancer cells resident in or transiting through the draining LN would probably provide considerable long-term therapeutic benefits to people with a wide variety of common cancers.

#### 5.1.1. Inhibitors of S1P Signaling and PI3K/Akt/mTOR Pathway

LNs constitute a critical crossroad between draining proteins, APCs, lymphocytes and even tumor cells. The tumor microenvironment including sentinel LNs is under immunosuppressive conditions such that the immune system is not able to eliminate cancer cells without immune-activating interventions. Constitutive activation or inhibition of various signaling pathways in human cancer cells can modulate multiple immunosuppressive cascades including cytokines, chemokines, and immunosuppressive cells [176]. Targeting S1P signaling leads to a significant suppression of cancer development in vivo, especially the inflammation-associated cancer [177]. In a syngeneic tumor model, administration of the specific sphingosine kinase 1 (SphK1) inhibitor suppresses S1P level, and reduces peritumoral lymphatic density and LN metastases [50]. LyP-1 peptide specifically binds to tumor cells and LECs in certain tumors, but not lymphatics in normal tissues. Systemic LyP-1 peptide treatment of mice xenografted with breast cancer cells inhibits tumor growth characterized with foci of apoptotic cells and lack of lymphatics [159]. LyP-1-conjugated doxorubicin-loaded liposomes destroy tumor lymphatics and then inhibit LN metastases [178]. Bone morphogenetic protein 9 (BMP9) induces the dedifferentiation of LECs to BECs through a possible reduction in the transcription factor Prox-1 expression, and controls lymphatic maturation and valve formation during embryogenesis [160,179]. During tumorigenesis, bone morphogenetic protein 9 (BMP-9)/activin receptor-like kinase 1 (ALK-1) signals negatively regulate lymphatic formation [160], suggesting that BMP-9/ALK-1 signals have potential as a target for cancer therapy. The development of inhibitors to specifically target the PI3K/Akt/mTOR pathway may also represent a strategy for the prevention and treatment of cancer. In a murine orthotopic model of head and neck SCC, inhibition of mTOR with rapamycin can diminish lymphangiogenesis in the primary tumors and prevent the dissemination of cancer cells to cervical LNs, thereby prolonging animal survival [161]. In patients, targeting mTOR is efficacious against advanced medullary thyroid carcinoma [162]. Recently, rapamycin inhibition of Freund's complete adjuvant (CFA)-induced lymphangiogenesis in popliteal LNs was found to be independent of mast cells [180]. Actually, the dual PI3K and mTOR inhibitors are either in clinical trials or already approved for treatment of patients.

#### 5.1.2. DC-Based Immunotherapy

CCL19 and CCL21 guide DC emigration out of tissues and immigration into the draining LN. The CCR7-CCL19/CCL21 axis is simultaneously involved in cancer cell dissemination and metastasis formation as well as in adaptive immune cell homing to lymphoid organs. Accumulated DCs in LNs can activate naïve T cells, determining the therapeutic effects of DC-based immunotherapy [181]. In this context, the development of immune-based clinical implications for cancer treatment should be taken into account when interpreting the significance of LN-LEC immune functions. Metastatic cells often reach the LNs by mimicking the molecular mechanisms used by hematopoietic cells to traffic to peripheral lymphoid organs. On the basis of the theoretical opinion, oncolytic virotherapy and immunotherapy were performed by adoptive transfer of normal T cells loaded with oncolytic virus into tumor-bearing mice and virus-immune mice. The simple therapeutic treatment can both reduce the distribution of metastases and vaccinate the affected individual in situ against different cancer types [182]. Chemotherapy with cisplatin has induced activation and migration of antigen-loaded DCs into tumor-draining LNs dependent on the type I IFN pathway. Antigen density within the tumor is considered to be an important determinant of the outcome of immune surveillance following chemotherapy [183]. Clinical evaluation of IL-7 in human cancer is based on enhancement of adaptive immunity for improving T-cell survival and numbers, as well as T-cell repertoire diversity [163]. Administration of IL-7 has immune-activating effects by supporting lymph drainage function and thereby increasing the transport of antigen to draining LNs. However, the IL-7 therapy might enhance tumor lymphangiogenesis and metastatic spread in cancers [55].

#### 5.1.3. LN Transplantation and VEGF Inhibitors

LNs provide an immunological barrier against systemic dissemination of cancer cells and other pathogens. However, a number of tumors initially metastasize by spreading predominantly into regional LNs, where malignant cells grow indefinitely and without order. Effective LN transplantation holds potential for immunotherapy applications in the treatment of cancer and chronic infections. The outcome of LN transplantation has included functional reconstitution of the immunological barrier against tumor metastasis, which can be improved by subsequent administration of growth factors [184]. Actually, adenoviral VEGF-C or adenoviral VEGF-D therapy has greatly improved survival and functionality of transferred LNs by increasing lymphatic number and promoting lymph drainage [164]. Within tumor-bearing LNs, VEGF-A/C-mediated lymphangiogenesis results in increased lymph flow and lymphatic formation in distant LNs [185]. In experimental tumors, overexpression of VEGF-C and VEGF-D induces tumor lymphangiogenesis and promotes tumor metastasis, which can be inhibited by blocking the interaction of VEGFR-3 with its ligands [165,166]. Furthermore, COX-2 has been shown to upregulate VEGF-C and VEGF-D. The physiologic COX-2-dependent lymphangiogenesis usually occurs in the postpartum mammary gland. The increased peritumoral lymphatic density in human postpartum breast cancers, was found to contribute to the overall poor prognosis by promoting LN metastasis [167], suggesting that further study of the therapeutic efficacy of COX-2 inhibitors in cancer is warranted. In an experimental study, the direct contribution of tumor TIE-2-expressing monocytes and VEGFR kinase activities to the breast tumor lymphatic network was reported, suggesting a combined use of TIE-2 and VEGFR kinase inhibitors as a therapeutic approach to block lymphangiogenesis in breast cancer [186].

#### 5.1.4. Other Metastatic Inhibitors

TGF-β is a known inducer of integrin expression by tumor cells, and increased integrin-β3 is dependent adhesion of non-small-cell lung cancer cells to LECs, which are contributing to cancer metastatic spread. The dual targeting of TGF-β and integrin-β3 was reported to be used as a promising therapeutic approach to reduce lung cancer metastasis to LNs [124]. A recent experiment has also revealed that TGF-β-induced protein increases CCL21 expression in LECs, and anti-integrin β3 antibody inhibits lymphatic sprouting induced by the protein. In vivo inhibition of TGF-β-induced protein expression can greatly reduce tumor lymphangiogenesis and metastasis [117]. The activation of TGF-β-activated protein kinase 1 (TAK1) may up-regulate CCR7 expression and increase lymphatic invasion ability of cancer cells. Inhibition of TAK1 was found to decrease LN invasion and distant metastasis [168]. The role of neuropilin-2 as a potential therapeutic target in preventing LN metastasis has been highlighted by the fact that colorectal carcinoma can induce activation of neuropilin-2 in LECs to promote tumor lymphangiogenesis via integrin-α9β1/focal adhesion kinase (FAK)/Erk pathway independent VEGF-C/VEGFR-3 signaling [169]. Indeed, neuropilin-2 is semaphorin 3C and 3F receptor. Full-length semaphorin-3C was found to be an inhibitor of tumor lymphangiogenesis and metastasis, which can induce the collapse of LEC cytoskeleton in a neuropilin-2-dependent manner and inhibit VEGF-C-induced signal transduction and LEC proliferation [170]. Similarly, recombinant semaphorin 3F also promotes LEC collapse and potently inhibits lymphangiogenesis in vivo and may represent an antilymphangiogenic metastasis suppressor gene in head and neck squamous cell carcinomas (HNSCC) [171]. Tumor-secreted adrenomedullin is a critical factor for inducing lymphangiogenesis and lymphatic enlargement through a pharmacologically tractable G-protein-coupled receptor both in primary tumor tissues and sentinel LNs, suggesting targeting of adrenomedullin signaling may provide a new avenue for inhibiting tumor progress [187].

The nanoparticles depend on lymph drainage to reach the LNs, and target different cells within a given organ, which can substantially affect the quality of the immunological response by interacting with monocytes, macrophages and myeloid DCs [188]. The anticancer efficacy of a supramolecular complex was reported to be used as an artificial enzyme against multi-drug-resistant cancer cells. Tumor actively-targeted theranostic nanoparticles have been developed for therapeutic and diagnostic applications [136,189]. In addition, despite small-molecule drugs, biologics, miRNA, RNA interference (RNAi) and vaccines are under active investigation, the cancer stem cell (CSCs) involved in tumor-induced lymphangiogenesis are now emerging as a plausible target for new drug discovery [190,191]. CSC model may provide a theoretical basis for developing therapies that target the minority CSC population, and present a new perspective for the treatment of cancer [192]. Obviously, novel approaches for inhibiting LN metastasis may offer the advantage of specific tumor targeting, limiting healthy tissue exposure and toxicity. Targeted therapies are currently the focus of much anticancer drug development, which may control LN lymphangiogenesis and tumor cell adhesion to LN-LECs.

### 5.2. Perspectives

Significant advances have been made in the last decade to our understanding of lymphatic involvement in lymphedema, inflammation and cancer metastasis. Most of them have uncovered previously unseen phenomena by utilizing innovative techniques to visualize the change of lymphatic structures and functions in a wide variety of transgenic and knockout models and in human diseases.

LECs are involved in cancer metastasis by facilitating recruitment of tumor cells into initial lymphatics and draining LNs, by creating a cancer stem cell niche to enable tumor progression, and by regulating host antitumor immune responses to potentially improve clinical outcomes [193]. Lymphangiogenesis in primary tumors and LNs represents an important prognostic marker for the risk of future metastasis and overall survival in many kinds of cancers [101]. An increased number and size of peritumoral lymphatics may provide more opportunities for cancer invasion and metastasis, and increased pumping and lymph flow may create a dynamic force for accelerating cancer spreading to the draining LNs, especially in the majority of epithelial cancers [77,154]. Emerging evidence has suggested that LN-LECs play a novel role in regulating host immunity, including dampening DC maturation and secreting immunosuppressive factors for suppression of T-cell function. LN-LECs may drive T-cell tolerance by antigen presentation, and actively scavenge exogenous lymph-borne antigens or regulate transendothelial transport [70,142]. In fact, the intralymphatic malignant cells may fail to reach the sentinel LN possibly by host immunity, or gain access to the lymphatics as one of the earliest metastatic events before sentinel LNs are positive, or is present in the regional LN basin but undetectable [194]. Thus, it remains to be addressed whether a significant correlation exists between lymphatic invasion and sentinel LN involvement, and how the metastatic cells exit from LNs. Furthermore, in tumor microenvironment, what kind of differences of lymphatic phenotypes and functions are there between the sentinel and non-sentinel LNs? How does the sentinel LN act as a barrier or facilitator during cancer progression? In the near future, inducible labeling of malignant cells for entering and exiting from sentinel LNs holds great promise to develop new and innovative treatments for cancer [92].

The relevance of lymphangiogenesis in tumor-induced immune modulation of sentinel LNs and premetastatic niche formation still remains an open question. The premetastatic niche in LNs represents a specialized microenvironment that promotes the survival and outgrowth of disseminated tumor cells. What are the cells involved in the niche, and what are their morphologic, immunophenotypic, and molecular features? Is it possible to reprogram the premetastatic niche cells to alter premetastatic microenvironment. Understanding cellular and molecular mechanisms regulating intranodal lymphatic reconstruction and remodeling should be a prerequisite to novel therapeutic approaches for human cancers. The strategy for targeting premetastatic niche molecules may prevent the spread of cancer cells to distant sites. However, considering molecular targeted therapy of cancer, unraveling and exploiting the involvement of LN-LECs in immune response stimulation and tolerance continues to be a challenging task.

Therefore, the research strategy involves the convergence of multiple fields such as lymphatic biology, immunology, bioengineering, and tumor microenvironment. The most relevant questions and approaches should be defined to help answer if biological and immunological issues in the interaction of tumor cells and LN-LECs hold the key to the design of successful anti-cancer therapeutics. In this respect, there is a growing need for the identification of molecules with particular biological effects in cell-based assays or animal models, to evaluate the functional status of tumor-associated lymphatics and to provide a quantitative framework for guiding preclinical trials of new anticancer chemotherapies, targeted therapies and even immunotherapies.

## 6. Conclusions

A dynamic process in the LN homeostasis and remodeling involves different cellular and molecular components, and T cell/B cell zones. Increasing evidence supports a novel functional role of LN-LECs in immune response through producing cytokines and chemokines. LN metastasis is involved in regulation by multiple factors and different cell types, which drive the migration of tumor cells into lymphatics and beyond, and regulate the balance between host immunity and tolerance. Induction of lymphangiogenesis by VEGF-A/-C and increased interstitial flow are positively correlated with cancer cell dissemination to the draining LNs. Tumor-derived VEGF-C induces lymphatic hyperplasia in sentinel LNs even before tumor metastasis, involved in a formation of premetastatic niche. Intranodal lymphatic sinuses are also greatly influenced by CCR7-CCL19/CCL21, CXCL12/CXCR4, S1P, IL-7, IFN-γ, integrin α4β1, and TGF-β in tumor microenvironment. Therefore, a greater understanding of the cellular and molecular control and modulation of LN-LECs may have therapeutic value in the treatment of cancers.

## Figures and Tables

**Table 1:** Molecules and factors relating to the structure and immune function of lymph nodes (LNs) and intranodal lymphatic endothelial cells (LECs).

Division	Molecules and Factors	Function and Property	References
Regulation of Cell Trafficking	CCL19/CCL21/CCR7	Mainly regulating T cell and DC trafficking into LNs	[16,33]
	CXCL12 (SDF-1)/CXCR4; CXCL13/CXCR5	Mainly regulating B cell trafficking into LNs (B cell homing)	
	S1P	Egress of lymphocytes from LNs into efferent lymphatics; Maturation of lymphatic intercellular junctions	[47,48]
LN Remodeling and Reconstruction	IL-7/IL-7Rα	LN organogenesis, development and maturation; Lymphocyte dynamics and homeostasis; Formation of lymphatic structures and morphological alteration	[30,53,54,55]
	LTβR	LN formation, homeostasis and remodeling; Regulating functions of intranodal lymphatic sinuses and HEVs	[39,59]
	CLEC-2	LN expansion, development and microarchitecture; Maintaining HEV integrity	[63,64,65,66]
Immune-Related Factors of LN-LECs	IFN-γ	Initiation of cell-mediated adaptive immune response; T cell proliferation and differentiation; T cell-mediated negative regulation of LN lymphangiogenesis	[18,67]
	VEGF-A	LN lymphangiogenesis, hypertrophy and HEV growth; T cell development, lymphocyte migration; DC mobilization and maturation; Antigen clearance and inflammation resolution	[21,68,69]
	VEGF-C	Suppression of antitumor immunity; Increasing dysfunctional activation of CD8 T cells	[70]
	CCL21	Ensuring lymph sampling and increase in lymph flow; Regulation of immunity and tolerance	[71,72,73]
	D6	Preventing inappropriate inflammatory leukocyte adherence to LECs and recruitment to LNs; Integration of innate and adaptive immune responses; Regulation of lymph flow	[74,75]
	PD-L1	LEC-induced peripheral tolerance	[76]

CCL19, chemokine (C–C motif) ligand 19; CCL21, chemokine (C–C motif) ligand 21; CCR7, chemokine (C–C motif) receptor 7; CXCL12, chemokine (C–X–C motif) ligand 12; CXCR4, chemokine (C–X–C motif) receptor 4; CXCL13, chemokine (C–X–C motif) ligand 13; CXCR5, chemokine (C–X–C motif) receptor 5; SDF-1, stromal cell-derived factor 1; S1P, lysophospholipid sphingosine-1-phosphate; IL7, interleukin 7; IL-7Rα, interleukin 7 receptor α (CD127); LTβR, lymphotoxin-β receptor; CLEC-2, C-type lectin-like receptor 2; IFN-γ, interferon-γ; VEGF-A/-C/-D, vascular endothelial growth factor-A/-C/-D; D6, chemokine-scavenging decoy receptor; PD-L1, programmed cell death ligand 1; LN, lymph node; LECs, lymphatic endothelial cells; HEVs, high endothelial venules; DCs, dendritic cells.

**Table 2:** Molecules and factors relating to intranodal LECs and LN metastasis.

Division	Molecules and Factors	Function and Property	References
LEC-Tumor Cell Interface and Prometastatic Factors	TGF-β	Tumor lymphangiogenesis and extracellular matrix formation; Lymphatic invasion and metastasis via EMT activation	[120,125]
	SIX-1	Promoting tumor lymphangiogenesis and LN metastasis via upregulation of TGF-β and VEGF-C expression, and EMT activation	[121,122,123]
	CCL19/CCL21/CCR7	LN metastatic dissemination of malignant cells; Lymphatic spread by recruiting tumor cells to T cell zone	[130,131]
	CCL1-CCR8	Mediating entry of tumor cells into LNs; TNF, IL-1β and lipopolysaccharide increase CCL1 production by LECs	[133]
	CXCL12 (SDF-1)/CXCR4	Controlling tumor metastasis by a lymphatic premetastatic niche	[135]
	IL-1	Promotion of lymphangiogenesis and LN metastasis through M2-type macrophages	[140]
	Integrin, ICAM-1, VCAM-1	Regulation of vascular stability, permeability, leukocyte migration and valve formation; Promotion of LN metastasis by adhesion of tumor cells to LN-LECs, and by establishment of a metastatic niche in LNs	[40,124,141,142]
	TLRs	Heterogeneous expression in LECs derived from different tissues; TLR deficiency is involved in decreased lymphangiogenesis and macrophage infiltration, and abnormal lymphatic architecture; Tumor progression and immune responses; Induction of prometastatic inflammatory response	[145,146,147,148,149]
	PPP2R1A-PPP2R1A homodimers	Expression on tumor cells and LECs; Regulation of cell-cell interactions at the lymphatic-tumor interface	[150]
LN Lymphangiogenesis and Metastasis	VEGF-C	Abnormal, nonfunctioning or immature lymphatic formation; Promotion of tumor cell survival inside LNs and entry into afferent lymphatics; Increased lymph flow	[21,153,154]
	EMILIN-1	Regulation of tumor phenotype and dormancy; Promotion of premetastatic niche formation and LN Invasion	[155]
	Apelin	Accelerated tumor growth; Increased intratumoral lymphangiogenesis	[156]
	Erythropoietin	Increase of VEGF-C expression in LN macrophages; Increase of LN lymphangiogenesis and nodal metastasis	[157]
	Prostaglandin	LN lymphangiogenesis; Induction of premetastatic niche formation	[158]
Inhibitors of Lymphatic Metastasis	SphK1 inhibitor	Suppressing lymphangiogenesis in tumor tissues and draining LNs; Suppressing S1P levels and tumor metastases to LNs	[50]
	LyP-1	Inhibiting tumor growth; Reduction of tumor lymphatic numbers	[159]
	BMP-9	Inhibition of lymphatic formation during tumorigenesis; Induction of dedifferentiation of LECs to BECs by reduction of *Prox-1* expression	[160]
	mTOR inhibitors	Reduction of tumor lymphangiogenesis; Prevention of cancer cell dissemination to LNs	[161,162]
	IL-7	Induction of lymphangiogenesis; Improvement of T-cell survival, numbers and repertoire diversity; Promotion of lymph drainage and antigen transport	[55,163]
	AdVEGF-C, AdVEGF-D	Improvement of survival and functionality of transferred LNs; Increase of lymphatic numbers; Promotion of lymph drainage	[164]
	Blockade of VEGF receptors	Anti-lymphangiogenic therapies; Inhibiting tumor metastasis	[165]
	COX-2 inhibitors	Inhibiting tumor lymphangiogenesis and metastasis	[166,167]
	TGF-β inhibitors	Reduction of tumor lymphangiogenesis and LN invasion	[117,168]
	Neuropilin-2 inhibitors, Recombinant semaphorin-3C/-3F	Promoting LEC collapse and inhibiting lymphangiogenesis	[169,170,171]

TGF-β, transforming growth factor-β; SIX-1, sine oculis homeobox homolog 1; CCL19, chemokine (C–C motif) ligand 19; CCL21, chemokine (C–C motif) ligand 21; CCR7, chemokine (C–C motif) receptor 7; CCL1, chemokine (C–C motif) ligand 1; CCR8, chemokine (C–C motif) receptor 8; CXCL12, chemokine (C–X–C motif) ligand 12; CXCR4, chemokine (C–X–C motif) receptor 4; SDF-1, stromal cell-derived factor 1; IL-1, interleukin-1; ICAM-1, intercellular adhesion molecule 1; VCAM-1, vascular cell adhesion molecule 1; TLRs, Toll-like receptors; PPP2R1A, protein phosphatase 2 regulatory subunit A, α-isoform; EMILIN-1, elastin microfibril interface-located protein 1; SphK1, sphingosine kinase 1; LyP-1, lymphatic peptide 1; BMP-9, bone morphogenetic protein 9; mTOR, mammalian target of rapamycin; IL7, interleukin 7; AdVEGF-C/-D, adenoviral VEGF-C/-D; COX-2, cyclooxygenase-2; LN, lymph node; LECs, lymphatic endothelial cells; EMT, epithelial-mesenchymal transition; BECs, blood vascular endothelial cells; Prox-1, prospero-related homeobox 1; TNF, tumor necrosis factor.

## References

[1] Ruddle N.H. (2014). Lymphatic vessels and tertiary lymphoid organs. J. Clin. Investig..

[2] Swartz M.A., Iida N., Roberts E.W., Sangaletti S., Wong M.H., Yull F.E., Coussens L.M., DeClerck Y.A. (2012). Tumor microenvironment complexity: Emerging roles in cancer therapy. Cancer Res..

[3] Lee M., Kiefel H., LaJevic M.D., Macauley M.S., Kawashima H., O’Hara E., Pan J., Paulson J.C., Butcher E.C. (2014). Transcriptional programs of lymphoid tissue capillary and high endothelium reveal control mechanisms for lymphocyte homing. Nat. Immunol..

[4] Thaunat O., Kerjaschki D., Nicoletti A. (2006). Is defective lymphatic drainage a trigger for lymphoid neogenesis?. Trends Immunol..

[5] Zhu M., Fu Y.X. (2011). The role of core TNF/LIGHT family members in lymph node homeostasis and remodeling. Immunol. Rev..

[6] Ji R.C. (2009). Lymph node lymphangiogenesis: A new concept for modulating tumor metastasis and inflammatory process. Histol. Histopathol..

[7] Lund A.W., Wagner M., Fankhauser M., Steinskog E.S., Broggi M.A., Spranger S., Gajewski T.F., Alitalo K., Eikesdal H.P., Wiig H. (2016). Lymphatic vessels regulate immune microenvironments in human and murine melanoma. J. Clin. Investig..

[8] Tauchi Y., Tanaka H., Kumamoto K., Tokumoto M., Sakimura C., Sakurai K., Kimura K., Toyokawa T., Amano R., Kubo N. (2016). Tumor-associated macrophages induce capillary morphogenesis of lymphatic endothelial cells derived from human gastric cancer. Cancer Sci..

[9] Morton D.L., Thompson J.F., Cochran A.J., Mozzillo N., Nieweg O.E., Roses D.F., Hoekstra H.J., Karakousis C.P., Puleo C.A., Coventry B.J. (2014). Final trial report of sentinel-node biopsy versus nodal observation in melanoma. N. Engl. J. Med..

[10] Hirosue S., Vokali E., Raghavan V.R., Rincon-Restrepo M., Lund A.W., Corthésy-Henrioud P., Capotosti F., Winter C.H., Hugues S., Swartz M.A. (2014). Steady-state antigen scavenging, cross-presentation, and CD8^+^ T cell priming: A new role for lymphatic endothelial cells. J. Immunol..

[11] Podgrabinska S., Skobe M. (2014). Role of lymphatic vasculature in regional and distant metastases. Microvasc. Res..

[12] Card C.M., Yu S.S., Swartz M.A. (2014). Emerging roles of lymphatic endothelium in regulating adaptive immunity. J. Clin. Investig..

[13] Ji R.C. (2014). Hypoxia and lymphangiogenesis in tumor microenvironment and metastasis. Cancer Lett..

[14] Drayton D.L., Liao S., Mounzer R.H., Ruddle N.H. (2006). Lymphoid organ development: From ontogeny to neogenesis. Nat. Immunol..

[15] Grigorova I.L., Panteleev M., Cyster J.G. (2010). Lymph node cortical sinus organization and relationship to lymphocyte egress dynamics and antigen exposure. Proc. Natl. Acad. Sci. USA.

[16] Link A., Vogt T.K., Favre S., Britschgi M.R., Acha-Orbea H., Hinz B., Cyster J.G., Luther S.A. (2007). Fibroblastic reticular cells in lymph nodes regulate the homeostasis of naive T cells. Nat. Immunol..

[17] Lukacs-Kornek V., Malhotra D., Fletcher A.L., Acton S.E., Elpek K.G., Tayalia P., Collier A.R., Turley S.J. (2011). Regulated release of nitric oxide by nonhematopoietic stroma controls expansion of the activated T cell pool in lymph nodes. Nat. Immunol..

[18] Kataru R.P., Kim H., Jang C., Choi D.K., Koh B.I., Kim M., Gollamudi S., Kim Y.K., Lee S.H., Koh G.Y. (2011). T lymphocytes negatively regulate lymph node lymphatic vessel formation. Immunity.

[19] Sixt M., Kanazawa N., Selg M., Samson T., Roos G., Reinhardt D.P., Pabst R., Lutz M.B., Sorokin L. (2005). The conduit system transports soluble antigens from the afferent lymph to resident dendritic cells in the T cell area of the lymph node. Immunity.

[20] Park S.M., Angel C.E., McIntosh J.D., Mansell C.M., Chen C.J., Cebon J., Dunbar P.R. (2014). Mapping the distinctive populations of lymphatic endothelial cells in different zones of human lymph nodes. PLoS ONE.

[21] Kataru R.P., Jung K., Jang C., Yang H., Schwendener R.A., Baik J.E., Han S.H., Alitalo K., Koh G.Y. (2009). Critical role of CD11b^+^ macrophages and VEGF in inflammatory lymphangiogenesis, antigen clearance, and inflammation resolution. Blood.

[22] Randolph G.J., Angeli V., Swartz M.A. (2005). Dendritic-cell trafficking to lymph nodes through lymphatic vessels. Nat. Rev. Immunol..

[23] Kesler C.T., Liao S., Munn L.L., Padera T.P. (2013). Lymphatic vessels in health and disease. Wiley Interdiscip. Rev. Syst. Biol. Med..

[24] Bajénoff M., Germain R.N. (2009). B-cell follicle development remodels the conduit system and allows soluble antigen delivery to follicular dendritic cells. Blood.

[25] Fletcher A.L., Malhotra D., Turley S.J. (2011). Lymph node stroma broaden the peripheral tolerance paradigm. Trends Immunol..

[26] Katakai T., Hara T., Sugai M., Gonda H., Shimizu A. (2004). Lymph node fibroblastic reticular cells construct the stromal reticulum via contact with lymphocytes. J. Exp. Med..

[27] Gretz J.E., Norbury C.C., Anderson A.O., Proudfoot A.E., Shaw S. (2000). Lymph-borne chemokines and other low molecular weight molecules reach high endothelial venules via specialized conduits while a functional barrier limits access to the lymphocyte microenvironments in lymph node cortex. J. Exp. Med..

[28] Braun A., Worbs T., Moschovakis G.L., Halle S., Hoffmann K., Bölter J., Münk A., Förster R. (2011). Afferent lymph-derived T cells and DCs use different chemokine receptor CCR7-dependent routes for entry into the lymph node and intranodal migration. Nat. Immunol..

[29] Muñoz-Fernández R., Blanco F.J., Frecha C., Martín F., Kimatrai M., Abadía-Molina A.C., García-Pacheco J.M., Olivares E.G. (2006). Follicular dendritic cells are related to bone marrow stromal cell progenitors and to myofibroblasts. J. Immunol..

[30] Onder L., Narang P., Scandella E., Chai Q., Iolyeva M., Hoorweg K., Halin C., Richie E., Kaye P., Westermann J. (2012). IL-7-producing stromal cells are critical for lymph node remodeling. Blood.

[31] Stacker S.A., Williams S.P., Karnezis T., Shayan R., Fox S.B., Achen M.G. (2014). Lymphangiogenesis and lymphatic vessel remodelling in cancer. Nat. Rev. Cancer.

[32] Cyster J.G., Schwab S.R. (2012). Sphingosine-1-phosphate and lymphocyte egress from lymphoid organs. Annu. Rev. Immunol..

[33] Luther S.A., Bidgol A., Hargreaves D.C., Schmidt A., Xu Y., Paniyadi J., Matloubian M., Cyster J.G. (2002). Differing activities of homeostatic chemokines CCL19, CCL21, and CXCL12 in lymphocyte and dendritic cell recruitment and lymphoid neogenesis. J. Immunol..

[34] MartIn-Fontecha A., Sebastiani S., Höpken U.E., Uguccioni M., Lipp M., Lanzavecchia A., Sallusto F. (2003). Regulation of dendritic cell migration to the draining lymph node: Impact on T lymphocyte traffic and priming. J. Exp. Med..

[35] Förster R., Davalos-Misslitz A.C., Rot A. (2008). CCR7 and its ligands: Balancing immunity and tolerance. Nat. Rev. Immunol..

[36] Weber M., Hauschild R., Schwarz J., Moussion C., de Vries I., Legler D.F., Luther S.A., Bollenbach T., Sixt M. (2013). Interstitial dendritic cell guidance by haptotactic chemokine gradients. Science.

[37] Ulvmar M.H., Werth K., Braun A., Kelay P., Hub E., Eller K., Chan L., Lucas B., Novitzky-Basso I., Nakamura K. (2014). The atypical chemokine receptor CCRL1 shapes functional CCL21 gradients in lymph nodes. Nat. Immunol..

[38] Moussion C., Girard J.P. (2011). Dendritic cells control lymphocyte entry to lymph nodes through high endothelial venules. Nature.

[39] Onder L., Danuser R., Scandella E., Firner S., Chai Q., Hehlgans T., Stein J.V., Ludewig B. (2013). Endothelial cell-specific lymphotoxin-β receptor signaling is critical for lymph node and high endothelial venule formation. J. Exp. Med..

[40] Bazigou E., Lyons O.T., Smith A., Venn G.E., Cope C., Brown N.A., Makinen T. (2011). Genes regulating lymphangiogenesis control venous valve formation and maintenance in mice. J. Clin. Investig..

[41] Murtomaki A., Uh M.K., Kitajewski C., Zhao J., Nagasaki T., Shawber C.J., Kitajewski J. (2014). Notch signaling functions in lymphatic valve formation. Development.

[42] Mishima K., Watabe T., Saito A., Yoshimatsu Y., Imaizumi N., Masui S., Hirashima M., Morisada T., Oike Y., Araie M. (2007). Prox1 induces lymphatic endothelial differentiation via integrin α9 and other signaling cascades. Mol. Biol. Cell.

[43] Ito K., Morimoto J., Kihara A., Matsui Y., Kurotaki D., Kanayama M., Simmons S., Ishii M., Sheppard D., Takaoka A. (2014). Integrin α9 on lymphatic endothelial cells regulates lymphocyte egress. Proc. Natl. Acad. Sci. USA.

[44] Karikoski M., Marttila-Ichihara F., Elima K., Rantakari P., Hollmén M., Kelkka T., Gerke H., Huovinen V., Irjala H., Holmdahl R. (2014). Clever-1/stabilin-1 controls cancer growth and metastasis. Clin. Cancer Res..

[45] Irjala H., Johansson E.L., Grenman R., Alanen K., Salmi M., Jalkanen S. (2001). Mannose receptor is a novel ligand for l-selectin and mediates lymphocyte binding to lymphatic endothelium. J. Exp. Med..

[46] Marttila-Ichihara F., Turja R., Miiluniemi M., Karikoski M., Maksimow M., Niemelä J., Martinez-Pomares L., Salmi M., Jalkanen S. (2008). Macrophage mannose receptor on lymphatics controls cell trafficking. Blood.

[47] Pappu R., Schwab S.R., Cornelissen I., Pereira J.P., Regard J.B., Xu Y., Camerer E., Zheng Y.W., Huang Y., Cyster J.G. (2007). Promotion of lymphocyte egress into blood and lymph by distinct sources of sphingosine-1-phosphate. Science.

[48] Pham T.H., Baluk P., Xu Y., Grigorova I., Bankovich A.J., Pappu R., Coughlin S.R., McDonald D.M., Schwab S.R., Cyster J.G. (2010). Lymphatic endothelial cell sphingosine kinase activity is required for lymphocyte egress and lymphatic patterning. J. Exp. Med..

[49] Nagahashi M., Kim E.Y., Yamada A., Ramachandran S., Allegood J.C., Hait N.C., Maceyka M., Milstien S., Takabe K., Spiegel S. (2013). Spns2, a transporter of phosphorylated sphingoid bases, regulates their blood and lymph levels, and the lymphatic network. FASEB J..

[50] Nagahashi M., Ramachandran S., Kim E.Y., Allegood J.C., Rashid O.M., Yamada A., Zhao R., Milstien S., Zhou H., Spiegel S. (2012). Sphingosine-1-phosphate produced by sphingosine kinase 1 promotes breast cancer progression by stimulating angiogenesis and lymphangiogenesis. Cancer Res..

[51] Jang J.Y., Koh Y.J., Lee S.H., Lee J., Kim K.H., Kim D., Koh G.Y., Yoo O.J. (2013). Conditional ablation of LYVE-1^+^ cells unveils defensive roles of lymphatic vessels in intestine and lymph nodes. Blood.

[52] Webster B., Ekland E.H., Agle L.M., Chyou S., Ruggieri R., Lu T.T. (2006). Regulation of lymph node vascular growth by dendritic cells. J. Exp. Med..

[53] Hara T., Shitara S., Imai K., Miyachi H., Kitano S., Yao H., Tani-ichi S., Ikuta K. (2012). Identification of IL-7-producing cells in primary and secondary lymphoid organs using IL-7-GFP knock-in mice. J. Immunol..

[54] Coles M.C., Veiga-Fernandes H., Foster K.E., Norton T., Pagakis S.N., Seddon B., Kioussis D. (2006). Role of T and NK cells and IL7/IL7r interactions during neonatal maturation of lymph nodes. Proc. Natl. Acad. Sci. USA.

[55] Iolyeva M., Aebischer D., Proulx S.T., Willrodt A.H., Ecoiffier T., Häner S., Bouchaud G., Krieg C., Onder L., Ludewig B. (2013). Interleukin-7 is produced by afferent lymphatic vessels and supports lymphatic drainage. Blood.

[56] Meier D., Bornmann C., Chappaz S., Schmutz S., Otten L.A., Ceredig R., Acha-Orbea H., Finke D. (2007). Ectopic lymphoid-organ development occurs through interleukin 7-mediated enhanced survival of lymphoid-tissue-inducer cells. Immunity.

[57] Roozendaal R., Mebius R.E. (2011). Stromal cell-immune cell interactions. Annu. Rev. Immunol..

[58] Chappaz S., Finke D. (2010). The IL-7 signaling pathway regulates lymph node development independent of peripheral lymphocytes. J. Immunol..

[59] Liao S., Ruddle N.H. (2006). Synchrony of high endothelial venules and lymphatic vessels revealed by immunization. J. Immunol..

[60] Schneider K., Potter K.G., Ware C.F. (2004). Lymphotoxin and LIGHT signaling pathways and target genes. Immunol. Rev..

[61] Vondenhoff M.F., Greuter M., Goverse G., Elewaut D., Dewint P., Ware C.F., Hoorweg K., Kraal G., Mebius R.E. (2009). LTβR signaling induces cytokine expression and up-regulates lymphangiogenic factors in lymph node anlagen. J. Immunol..

[62] Lee S.Y., Chao-Nan Q., Seng O.A., Peiyi C., Bernice W.H., Swe M.S., Chii W.J., Jacqueline H.S., Chee S.K. (2012). Changes in specialized blood vessels in lymph nodes and their role in cancer metastasis. J. Transl. Med..

[63] Herzog B.H., Fu J., Wilson S.J., Hess P.R., Sen A., McDaniel J.M., Pan Y., Sheng M., Yago T., Silasi-Mansat R. (2013). Podoplanin maintains high endothelial venule integrity by interacting with platelet CLEC-2. Nature.

[64] Acton S.E., Astarita J.L., Malhotra D., Lukacs-Kornek V., Franz B., Hess P.R., Jakus Z., Kuligowski M., Fletcher A.L., Elpek K.G. (2012). Podoplanin-rich stromal networks induce dendritic cell motility via activation of the C-type lectin receptor CLEC-2. Immunity.

[65] Acton S.E., Farrugia A.J., Astarita J.L., Mourão-Sá D., Jenkins R.P., Nye E., Hooper S., van Blijswijk J., Rogers N.C., Snelgrove K.J. (2014). Dendritic cells control fibroblastic reticular network tension and lymph node expansion. Nature.

[66] Astarita J.L., Cremasco V., Fu J., Darnell M.C., Peck J.R., Nieves-Bonilla J.M., Song K., Kondo Y., Woodruff M.C., Gogineni A. (2015). The CLEC-2-podoplanin axis controls the contractility of fibroblastic reticular cells and lymph node microarchitecture. Nat. Immunol..

[67] Nörder M., Gutierrez M.G., Zicari S., Cervi E., Caruso A., Guzmán C.A. (2012). Lymph node-derived lymphatic endothelial cells express functional costimulatory molecules and impair dendritic cell-induced allogenic T-cell proliferation. FASEB J..

[68] Shrestha B., Hashiguchi T., Ito T., Miura N., Takenouchi K., Oyama Y., Kawahara K., Tancharoen S., Ki-I Y., Arimura N. (2010). B cell-derived vascular endothelial growth factor A promotes lymphangiogenesis and high endothelial venule expansion in lymph nodes. J. Immunol..

[69] Angeli V., Ginhoux F., Llodrà J., Quemeneur L., Frenette P.S., Skobe M., Jessberger R., Merad M., Randolph G.J. (2006). B cell-driven lymphangiogenesis in inflamed lymph nodes enhances dendritic cell mobilization. Immunity.

[70] Lund A.W., Duraes F.V., Hirosue S., Raghavan V.R., Nembrini C., Thomas S.N., Issa A., Hugues S., Swartz M.A. (2012). VEGF-C promotes immune tolerance in B16 melanomas and cross-presentation of tumor antigen by lymph node lymphatics. Cell Rep..

[71] Miteva D.O., Rutkowski J.M., Dixon J.B., Kilarski W., Shields J.D., Swartz M.A. (2010). Transmural flow modulates cell and fluid transport functions of lymphatic endothelium. Circ. Res..

[72] Tomei A.A., Siegert S., Britschgi M.R., Luther S.A., Swartz M.A. (2009). Fluid flow regulates stromal cell organization and CCL21 expression in a tissue-engineered lymph node microenvironment. J. Immunol..

[73] Shields J.D., Kourtis I.C., Tomei A.A., Roberts J.M., Swartz M.A. (2010). Induction of lymphoidlike stroma and immune escape by tumors that express the chemokine CCL21. Science.

[74] Fra A.M., Locati M., Otero K., Sironi M., Signorelli P., Massardi M.L., Gobbi M., Vecchi A., Sozzani S., Mantovani A. (2003). Cutting edge: Scavenging of inflammatory CC chemokines by the promiscuous putatively silent chemokine receptor D6. J. Immunol..

[75] Lee K.M., McKimmie C.S., Gilchrist D.S., Pallas K.J., Nibbs R.J., Garside P., McDonald V., Jenkins C., Ransohoff R., Liu L. (2011). D6 facilitates cellular migration and fluid flow to lymph nodes by suppressing lymphatic congestion. Blood.

[76] Tewalt E.F., Cohen J.N., Rouhani S.J., Guidi C.J., Qiao H., Fahl S.P., Conaway M.R., Bender T.P., Tung K.S., Vella A.T. (2012). Lymphatic endothelial cells induce tolerance via PD-L1 and lack of costimulation leading to high-level PD-1 expression on CD8 T cells. Blood.

[77] Harrell M.I., Iritani B.M., Ruddell A. (2007). Tumor-induced sentinel lymph node lymphangiogenesis and increased lymph flow precede melanoma metastasis. Am. J. Pathol..

[78] Kwon S., Agollah G.D., Wu G., Chan W., Sevick-Muraca E.M. (2013). Direct visualization of changes of lymphatic function and drainage pathways in lymph node metastasis of B16F10 melanoma using near-infrared fluorescence imaging. Biomed. Opt. Express.

[79] Ruddell A., Kelly-Spratt K.S., Furuya M., Parghi S.S., Kemp C.J. (2008). p19/Arf and p53 suppress sentinel lymph node lymphangiogenesis and carcinoma metastasis. Oncogene.

[80] Swartz M.A., Lund A.W. (2012). Lymphatic and interstitial flow in the tumour microenvironment: Linking mechanobiology with immunity. Nat. Rev. Cancer.

[81] Cohen J.N., Guidi C.J., Tewalt E.F., Qiao H., Rouhani S.J., Ruddell A., Farr A.G., Tung K.S., Engelhard V.H. (2010). Lymph node-resident lymphatic endothelial cells mediate peripheral tolerance via Aire-independent direct antigen presentation. J. Exp. Med..

[82] Tewalt E.F., Cohen J.N., Rouhani S.J., Engelhard V.H. (2012). Lymphatic endothelial cells—Key players in regulation of tolerance and immunity. Front Immunol..

[83] Thomas S.N., Rutkowski J.M., Pasquier M., Kuan E.L., Alitalo K., Randolph G.J., Swartz M.A. (2012). Impaired humoral immunity and tolerance in K14-VEGFR-3-Ig mice that lack dermal lymphatic drainage. J. Immunol..

[84] Liao S., Cheng G., Conner D.A., Huang Y., Kucherlapati R.S., Munn L.L., Ruddle N.H., Jain R.K., Fukumura D., Padera T.P. (2011). Impaired lymphatic contraction associated with immunosuppression. Proc. Natl. Acad. Sci. USA.

[85] Niedbala W., Cai B., Liu H., Pitman N., Chang L., Liew F.Y. (2007). Nitric oxide induces CD4^+^CD25^+^ Foxp3 regulatory T cells from CD4^+^CD25 T cells via p53, IL-2, and OX40. Proc. Natl. Acad. Sci. USA.

[86] Wirzenius M., Tammela T., Uutela M., He Y., Odorisio T., Zambruno G., Nagy J.A., Dvorak H.F., Ylä-Herttuala S., Shibuya M. (2007). Distinct vascular endothelial growth factor signals for lymphatic vessel enlargement and sprouting. J. Exp. Med..

[87] Yoo S.A., Bae D.G., Ryoo J.W., Kim H.R., Park G.S., Cho C.S., Chae C.B., Kim W.U. (2005). Arginine-rich anti-vascular endothelial growth factor (anti-VEGF) hexapeptide inhibits collagen-induced arthritis and VEGF-stimulated productions of TNF-α and IL-6 by human monocytes. J. Immunol..

[88] Nibbs R.J., Kriehuber E., Ponath P.D., Parent D., Qin S., Campbell J.D., Henderson A., Kerjaschki D., Maurer D., Graham G.J. (2001). The β-chemokine receptor D6 is expressed by lymphatic endothelium and a subset of vascular tumors. Am. J. Pathol..

[89] McKimmie C.S., Singh M.D., Hewit K., Lopez-Franco O., Le Brocq M., Rose-John S., Lee K.M., Baker A.H., Wheat R., Blackbourn D.J. (2013). An analysis of the function and expression of D6 on lymphatic endothelial cells. Blood.

[90] Gogineni A., Caunt M., Crow A., Lee C.V., Fuh G., van Bruggen N., Ye W., Weimer R.M. (2013). Inhibition of VEGF-C modulates distal lymphatic remodeling and secondary metastasis. PLoS ONE.

[91] Takeuchi H., Kitajima M., Kitagawa Y. (2008). Sentinel lymph node as a target of molecular diagnosis of lymphatic micrometastasis and local immunoresponse to malignant cells. Cancer Sci..

[92] Alitalo A., Detmar M. (2012). Interaction of tumor cells and lymphatic vessels in cancer progression. Oncogene.

[93] Liersch R., Hirakawa S., Berdel W.E., Mesters R.M., Detmar M. (2012). Induced lymphatic sinus hyperplasia in sentinel lymph nodes by VEGF-C as the earliest premetastatic indicator. Int. J. Oncol..

[94] Qian C.N., Berghuis B., Tsarfaty G., Bruch M., Kort E.J., Ditlev J., Tsarfaty I., Hudson E., Jackson D.G., Petillo D. (2006). Preparing the “soil”: The primary tumor induces vasculature reorganization in the sentinel lymph node before the arrival of metastatic cancer cells. Cancer Res..

[95] Mansfield A.S., Holtan S.G., Grotz T.E., Allred J.B., Jakub J.W., Erickson L.A., Markovic S.N. (2011). Regional immunity in melanoma: Immunosuppressive changes precede nodal metastasis. Mod. Pathol..

[96] Mohos A., Sebestyén T., Liszkay G., Plótár V., Horváth S., Gaudi I., Ladányi A. (2013). Immune cell profile of sentinel lymph nodes in patients with malignant melanoma—FOXP3^+^ cell density in cases with positive sentinel node status is associated with unfavorable clinical outcome. J. Transl. Med..

[97] Hirakawa S., Brown L.F., Kodama S., Paavonen K., Alitalo K., Detmar M. (2007). VEGF-C-induced lymphangiogenesis in sentinel lymph nodes promotes tumor metastasis to distant sites. Blood.

[98] Kerjaschki D., Bago-Horvath Z., Rudas M., Sexl V., Schneckenleithner C., Wolbank S., Bartel G., Krieger S., Kalt R., Hantusch B. (2011). Lipoxygenase mediates invasion of intrametastatic lymphatic vessels and propagates lymph node metastasis of human mammary carcinoma xenografts in mouse. J. Clin. Investig..

[99] Nakanishi M., Morita Y., Hata K., Muragaki Y. (2016). Acidic microenvironments induce lymphangiogenesis and IL-8 production via TRPV1 activation in human lymphatic endothelial cells. Exp. Cell Res..

[100] Ariffin A.B., Forde P.F., Jahangeer S., Soden D.M., Hinchion J. (2014). Releasing pressure in tumors: What do we know so far and where do we go from here? A review. Cancer Res..

[101] Ji R.C. (2006). Lymphatic endothelial cells, tumor lymphangiogenesis and metastasis: New insights into intratumoral and peritumoral lymphatics. Cancer Metastasis Rev..

[102] Ji R.C., Eshita Y., Kato S. (2007). Investigation of intratumoural and peritumoural lymphatics expressed by podoplanin and LYVE-1 in the hybridoma-induced tumours. Int. J. Exp. Pathol..

[103] Proulx S.T., Luciani P., Christiansen A., Karaman S., Blum K.S., Rinderknecht M., Leroux J.C., Detmar M. (2013). Use of a PEG-conjugated bright near-infrared dye for functional imaging of rerouting of tumor lymphatic drainage after sentinel lymph node metastasis. Biomaterials.

[104] Alitalo K. (2011). The lymphatic vasculature in disease. Nat. Med..

[105] Dadiani M., Kalchenko V., Yosepovich A., Margalit R., Hassid Y., Degani H., Seger D. (2006). Real-time imaging of lymphogenic metastasis in orthotopic human breast cancer. Cancer Res..

[106] Wang S., Zhang Q., Luo X.F., Li J., He H., Yang F., Di Y., Jin C., Jiang X.G., Shen S. (2014). Magnetic graphene-based nanotheranostic agent for dual-modality mapping guided photothermal therapy in regional lymph nodal metastasis of pancreatic cancer. Biomaterials.

[107] Wang Y., Lang L., Huang P., Wang Z., Jacobson O., Kiesewetter D.O., Ali I.U., Teng G., Niu G., Chen X. (2015). In vivo albumin labeling and lymphatic imaging. Proc. Natl. Acad. Sci. USA.

[108] Kerchner K., Fleischer A., Yosipovitch G. (2008). Lower extremity lymphedema update: Pathophysiology, diagnosis, and treatment guidelines. J. Am. Acad. Dermatol..

[109] Sevick-Muraca E.M., Kwon S., Rasmussen J.C. (2014). Emerging lymphatic imaging technologies for mouse and man. J. Clin. Investig..

[110] Yoo J.S., Lee S.C., Jow Z.Y., Koh P.Y., Chang Y.T. (2014). A macrophage-specific fluorescent probe for intraoperative lymph node staging. Cancer Res..

[111] Giampieri S., Manning C., Hooper S., Jones L., Hill C.S., Sahai E. (2009). Localized and reversible TGFβ signalling switches breast cancer cells from cohesive to single cell motility. Nat. Cell Biol..

[112] Chen Y.C., Allen S.G., Ingram P.N., Buckanovich R., Merajver S.D., Yoon E. (2015). Single-cell migration chip for chemotaxis-based microfluidic selection of heterogeneous cell populations. Sci. Rep..

[113] Heindl A., Nawaz S., Yuan Y. (2015). Mapping spatial heterogeneity in the tumor microenvironment: A new era for digital pathology. Lab. Investig..

[114] Kalluri R., Weinberg R.A. (2009). The basics of epithelial-mesenchymal transition. J. Clin. Investig..

[115] Thiery J.P., Acloque H., Huang R.Y., Nieto M.A. (2009). Epithelial-mesenchymal transitions in development and disease. Cell.

[116] Yang J., Mani S.A., Donaher J.L., Ramaswamy S., Itzykson R.A., Come C., Savagner P., Gitelman I., Richardson A., Weinberg R.A. (2004). Twist, a master regulator of morphogenesis, plays an essential role in tumor metastasis. Cell.

[117] Maeng Y.S., Aguilar B., Choi S.I., Kim E.K. (2016). Inhibition of TGFBIp expression reduces lymphangiogenesis and tumor metastasis. Oncogene.

[118] Xu J., Lamouille S., Derynck R. (2009). TGF-β-induced epithelial to mesenchymal transition. Cell Res..

[119] Fuxe J., Karlsson M.C. (2012). TGF-β-induced epithelial-mesenchymal transition: A link between cancer and inflammation. Semin. Cancer Biol..

[120] Pang M.F., Georgoudaki A.M., Lambut L., Johansson J., Tabor V., Hagikura K., Jin Y., Jansson M., Alexander J.S., Nelson C.M. (2016). TGF-β1-induced EMT promotes targeted migration of breast cancer cells through the lymphatic system by the activation of CCR7/CCL21-mediated chemotaxis. Oncogene.

[121] Micalizzi D.S., Christensen K.L., Jedlicka P., Coletta R.D., Barón A.E., Harrell J.C., Horwitz K.B., Billheimer D., Heichman K.A., Welm A.L. (2009). The Six1 homeoprotein induces human mammary carcinoma cells to undergo epithelial-mesenchymal transition and metastasis in mice through increasing TGF-β signaling. J. Clin. Investig..

[122] Wang C.A., Jedlicka P., Patrick A.N., Micalizzi D.S., Lemmer K.C., Deitsch E., Casás-Selves M., Harrell J.C., Ford H.L. (2012). SIX1 induces lymphangiogenesis and metastasis via upregulation of VEGF-C in mouse models of breast cancer. J. Clin. Investig..

[123] Liu D., Li L., Zhang X.X., Wan D.Y., Xi B.X., Hu Z., Ding W.C., Zhu D., Wang X.L., Wang W. (2014). SIX1 promotes tumor lymphangiogenesis by coordinating TGFβ signals that increase expression of VEGF-C. Cancer Res..

[124] Salvo E., Garasa S., Dotor J., Morales X., Peláez R., Altevogt P., Rouzaut A. (2014). Combined targeting of TGF-β1 and integrin β3 impairs lymph node metastasis in a mouse model of non-small-cell lung cancer. Mol. Cancer.

[125] Oka M., Iwata C., Suzuki H.I., Kiyono K., Morishita Y., Watabe T., Komuro A., Kano M.R., Miyazono K. (2008). Inhibition of endogenous TGF-β signaling enhances lymphangiogenesis. Blood.

[126] Suzuki Y., Ito Y., Mizuno M., Kinashi H., Sawai A., Noda Y., Mizuno T., Shimizu H., Fujita Y., Matsui K. (2012). Transforming growth factor-β induces vascular endothelial growth factor-C expression leading to lymphangiogenesis in rat unilateral ureteral obstruction. Kidney Int..

[127] Irino T., Takeuchi H., Matsuda S., Saikawa Y., Kawakubo H., Wada N., Takahashi T., Nakamura R., Fukuda K., Omori T. (2014). CC-Chemokine receptor CCR7: A key molecule for lymph node metastasis in esophageal squamous cell carcinoma. BMC Cancer.

[128] Nakamura T., Shinriki S., Jono H., Guo J., Ueda M., Hayashi M., Yamashita S., Zijlstra A., Nakayama H., Hiraki A. (2015). Intrinsic TGF-β2-triggered SDF-1-CXCR4 signaling axis is crucial for drug resistance and a slow-cycling state in bone marrow-disseminated tumor cells. Oncotarget.

[129] Houshmand P., Zlotnik A. (2003). Therapeutic applications in the chemokine superfamily. Curr. Opin. Chem. Biol..

[130] Jung J.I., Cho H.J., Jung Y.J., Kwon S.H., Her S., Choi S.S., Shin S.H., Lee K.W., Park J.H. (2015). High-fat diet-induced obesity increases lymphangiogenesis and lymph node metastasis in the B16F10 melanoma allograft model: Roles of adipocytes and M2-macrophages. Int. J. Cancer.

[131] Rehm A., Mensen A., Schradi K., Gerlach K., Wittstock S., Winter S., Büchner G., Dörken B., Lipp M., Höpken U.E. (2011). Cooperative function of CCR7 and lymphotoxin in the formation of a lymphoma-permissive niche within murine secondary lymphoid organs. Blood.

[132] Sperveslage J., Frank S., Heneweer C., Egberts J., Schniewind B., Buchholz M., Bergmann F., Giese N., Munding J., Hahn S.A. (2012). Lack of CCR7 expression is rate limiting for lymphatic spread of pancreatic ductal adenocarcinoma. Int. J. Cancer.

[133] Das S., Sarrou E., Podgrabinska S., Cassella M., Mungamuri S.K., Feirt N., Gordon R., Nagi C.S., Wang Y., Entenberg D. (2013). Tumor cell entry into the lymph node is controlled by CCL1 chemokine expressed by lymph node lymphatic sinuses. J. Exp. Med..

[134] Peng S.B., Zhang X., Paul D., Kays L.M., Gough W., Stewart J., Uhlik M.T., Chen Q., Hui Y.H., Zamek-Gliszczynski M.J. (2015). Identification of LY2510924, a novel cyclic peptide CXCR4 antagonist that exhibits antitumor activities in solid tumor and breast cancer metastatic models. Mol. Cancer Ther..

[135] Kim M., Koh Y.J., Kim K.E., Koh B.I., Nam D.H., Alitalo K., Kim I., Koh G.Y. (2010). CXCR4 signaling regulates metastasis of chemoresistant melanoma cells by a lymphatic metastatic niche. Cancer Res..

[136] Eshita Y., Ji R.C., Onishi M., Kobayashi T., Mizuno M., Yoshida J., Kubota N., Onishi Y. (2015). Medicinal facilities to B16F10 melanoma cells for distant metastasis control with a supramolecular complex by DEAE-dextran-MMA copolymer/paclitaxel. Drug Deliv. Transl. Res..

[137] Fang W., Ye L., Shen L., Cai J., Huang F., Wei Q., Fei X., Chen X., Guan H., Wang W. (2014). Tumor-associated macrophages promote the metastatic potential of thyroid papillary cancer by releasing CXCL8. Carcinogenesis.

[138] Ji R.C. (2012). Macrophages are important mediators of either tumor- or inflammation-induced lymphangiogenesis. Cell. Mol. Life Sci..

[139] Qian B.Z., Pollard J.W. (2010). Macrophage diversity enhances tumor progression and metastasis. Cell.

[140] Watari K., Shibata T., Kawahara A., Sata K., Nabeshima H., Shinoda A., Abe H., Azuma K., Murakami Y., Izumi H. (2014). Tumor-derived interleukin-1 promotes lymphangiogenesis and lymph node metastasis through M2-type macrophages. PLoS ONE.

[141] Garmy-Susini B., Avraamides C.J., Desgrosellier J.S., Schmid M.C., Foubert P., Ellies L.G., Lowy A.M., Blair S.L., Vandenberg S.R., Datnow B. (2013). PI3Kα activates integrin α4β1 to establish a metastatic niche in lymph nodes. Proc. Natl. Acad. Sci. USA.

[142] Podgrabinska S., Kamalu O., Mayer L., Shimaoka M., Snoeck H., Randolph G.J., Skobe M. (2009). Inflamed lymphatic endothelium suppresses dendritic cell maturation and function via Mac-1/ICAM-1-dependent mechanism. J. Immunol..

[143] Iolyeva M., Karaman S., Willrodt A.H., Weingartner S., Vigl B., Halin C. (2013). Novel role for ALCAM in lymphatic network formation and function. FASEB J..

[144] Song Q., Xu Y., Yang C., Chen Z., Jia C., Chen J., Zhang Y., Lai P., Fan X., Zhou X. (2014). miR-483-5p promotes invasion and metastasis of lung adenocarcinoma by targeting RhoGDI1 and ALCAM. Cancer Res..

[145] Zhang Y., Lu Y., Ma L., Cao X., Xiao J., Chen J., Jiao S., Gao Y., Liu C., Duan Z. (2014). Activation of vascular endothelial growth factor receptor-3 in macrophages restrains TLR4-NF-κB signaling and protects against endotoxin shock. Immunity.

[146] Zampell J.C., Elhadad S., Avraham T., Weitman E., Aschen S., Yan A., Mehrara B.J. (2012). Toll-like receptor deficiency worsens inflammation and lymphedema after lymphatic injury. Am. J. Physiol. Cell Physiol..

[147] Garrafa E., Imberti L., Tiberio G., Prandini A., Giulini S.M., Caimi L. (2011). Heterogeneous expression of toll-like receptors in lymphatic endothelial cells derived from different tissues. Immunol. Cell Biol..

[148] Fabbri M., Paone A., Calore F., Galli R., Gaudio E., Santhanam R., Lovat F., Fadda P., Mao C., Nuovo G.J. (2012). MicroRNAs bind to Toll-like receptors to induce prometastatic inflammatory response. Proc. Natl. Acad. Sci. USA.

[149] Fernandez-Garcia B., Eiró N., González-Reyes S., González L., Aguirre A., González L.O., del Casar J.M., García-Muñiz J.L., Vizoso F.J. (2014). Clinical significance of toll-like receptor 3, 4, and 9 in gastric cancer. J. Immunother..

[150] Christianson D.R., Dobroff A.S., Proneth B., Zurita A.J., Salameh A., Dondossola E., Makino J., Bologa C.G., Smith T.L., Yao V.J. (2015). Ligand-directed targeting of lymphatic vessels uncovers mechanistic insights in melanoma metastasis. Proc. Natl. Acad. Sci. USA.

[151] Rofstad E.K., Huang R., Galappathi K., Andersen L.M., Wegner C.S., Hauge A., Gaustad J.V., Simonsen T.G. (2016). Functional intratumoral lymphatics in patient-derived xenograft models of squamous cell carcinoma of the uterine cervix: Implications for lymph node metastasis. Oncotarget.

[152] Davis M.J., Rahbar E., Gashev A.A., Zawieja D.C., Moore J.E. (2011). Determinants of valve gating in collecting lymphatic vessels from rat mesentery. Am. J. Physiol. Heart Circ. Physiol..

[153] Quagliata L., Klusmeier S., Cremers N., Pytowski B., Harvey A., Pettis R.J., Thiele W., Sleeman J.P. (2014). Inhibition of VEGFR-3 activation in tumor-draining lymph nodes suppresses the outgrowth of lymph node metastases in the MT-450 syngeneic rat breast cancer model. Clin. Exp. Metastasis.

[154] Proulx S.T., Luciani P., Derzsi S., Rinderknecht M., Mumprecht V., Leroux J.C., Detmar M. (2010). Quantitative imaging of lymphatic function with liposomal indocyanine green. Cancer Res..

[155] Danussi C., Petrucco A., Wassermann B., Modica T.M., Pivetta E., del Bel Belluz L., Colombatti A., Spessotto P. (2012). An EMILIN1-negative microenvironment promotes tumor cell proliferation and lymph node invasion. Cancer Prev. Res. (Phila).

[156] Berta J., Hoda M.A., Laszlo V., Rozsas A., Garay T., Torok S., Grusch M., Berger W., Paku S., Renyi-Vamos F. (2014). Apelin promotes lymphangiogenesis and lymph node metastasis. Oncotarget.

[157] Lee A.S., Kim D.H., Lee J.E., Jung Y.J., Kang K.P., Lee S., Park S.K., Kwak J.Y., Lee S.Y., Lim S.T. (2011). Erythropoietin induces lymph node lymphangiogenesis and lymph node tumor metastasis. Cancer Res..

[158] Ogawa F., Amano H., Eshima K., Ito Y., Matsui Y., Hosono K., Kitasato H., Iyoda A., Iwabuchi K., Kumagai Y. (2014). Prostanoid induces premetastatic niche in regional lymph nodes. J. Clin. Investig..

[159] Laakkonen P., Akerman M.E., Biliran H., Yang M., Ferrer F., Karpanen T., Hoffman R.M., Ruoslahti E. (2004). Antitumor activity of a homing peptide that targets tumor lymphatics and tumor cells. Proc. Natl. Acad. Sci. USA.

[160] Yoshimatsu Y., Lee Y.G., Akatsu Y., Taguchi L., Suzuki H.I., Cunha S.I., Maruyama K., Suzuki Y., Yamazaki T., Katsura A. (2013). Bone morphogenetic protein-9 inhibits lymphatic vessel formation via activin receptor-like kinase 1 during development and cancer progression. Proc. Natl. Acad. Sci. USA.

[161] Patel V., Marsh C.A., Dorsam R.T., Mikelis C.M., Masedunskas A., Amornphimoltham P., Nathan C.A., Singh B., Weigert R., Molinolo A.A. (2011). Decreased lymphangiogenesis and lymph node metastasis by mTOR inhibition in head and neck cancer. Cancer Res..

[162] Tamburrino A., Molinolo A.A., Salerno P., Chernock R.D., Raffeld M., Xi L., Gutkind J.S., Moley J.F., Wells S.A., Santoro M. (2012). Activation of the mTOR pathway in primary medullary thyroid carcinoma and lymph node metastases. Clin. Cancer Res..

[163] Mackall C.L., Fry T.J., Gress R.E. (2011). Harnessing the biology of IL-7 for therapeutic application. Nat. Rev. Immunol..

[164] Lähteenvuo M., Honkonen K., Tervala T., Tammela T., Suominen E., Lähteenvuo J., Kholová I., Alitalo K., Ylä-Herttuala S., Saaristo A. (2011). Growth factor therapy and autologous lymph node transfer in lymphedema. Circulation.

[165] Skobe M., Hawighorst T., Jackson D.G., Prevo R., Janes L., Velasco P., Riccardi L., Alitalo K., Claffey K., Detmar M. (2001). Induction of tumor lymphangiogenesis by VEGF-C promotes breast cancer metastasis. Nat. Med..

[166] Karnezis T., Shayan R., Caesar C., Roufail S., Harris N.C., Ardipradja K., Zhang Y.F., Williams S.P., Farnsworth R.H., Chai M.G. (2012). VEGF-D promotes tumor metastasis by regulating prostaglandins produced by the collecting lymphatic endothelium. Cancer Cell.

[167] Lyons T.R., Borges V.F., Betts C.B., Guo Q., Kapoor P., Martinson H.A., Jindal S., Schedin P. (2014). Cyclooxygenase-2-dependent lymphangiogenesis promotes nodal metastasis of postpartum breast cancer. J. Clin. Investig..

[168] Huang H.L., Chiang C.H., Hung W.C., Hou M.F. (2015). Targeting of TGF-β-activated protein kinase 1 inhibits chemokine (C–C motif) receptor 7 expression, tumor growth and metastasis in breast cancer. Oncotarget.

[169] Ou J.J., Wei X., Peng Y., Zha L., Zhou R.B., Shi H., Zhou Q., Liang H.J. (2015). Neuropilin-2 mediates lymphangiogenesis of colorectal carcinoma via a VEGFC/VEGFR3 independent signaling. Cancer Lett..

[170] Mumblat Y., Kessler O., Ilan N., Neufeld G. (2015). Full-length semaphorin-3C is an inhibitor of tumor lymphangiogenesis and metastasis. Cancer Res..

[171] Doçi C.L., Mikelis C.M., Lionakis M.S., Molinolo A.A., Gutkind J.S. (2015). Genetic identification of SEMA3F as an antilymphangiogenic metastasis suppressor gene in head and neck squamous carcinoma. Cancer Res..

[172] Kim J.D., Kang Y., Kim J., Papangeli I., Kang H., Wu J., Park H., Nadelmann E., Rockson S.G., Chun H.J. (2014). Essential role of Apelin signaling during lymphatic development in zebrafish. Arterioscler. Thromb. Vasc. Biol..

[173] Ghose S., Min Y., Lin P.C. (2015). δ-Catenin activates Rho GTPase, promotes lymphangiogenesis and growth of tumor metastases. PLoS ONE.

[174] Jordan-Williams K.L., Ramanujam N., Farr A.G., Ruddell A. (2016). The lymphatic endothelial mCLCA1 antibody induces proliferation and growth of lymph node lymphatic sinuses. PLoS ONE.

[175] Agrawal N., Frederick M.J., Pickering C.R., Bettegowda C., Chang K., Li R.J., Fakhry C., Xie T.X., Zhang J., Wang J. (2011). Exome sequencing of head and neck squamous cell carcinoma reveals inactivating mutations in NOTCH1. Science.

[176] Kawakami Y., Yaguchi T., Sumimoto H., Kudo-Saito C., Tsukamoto N., Iwata-Kajihara T., Nakamura S., Nishio H., Satomi R., Kobayashi A. (2013). Cancer-induced immunosuppressive cascades and their reversal by molecular-targeted therapy. Ann. N. Y. Acad. Sci..

[177] Liang J., Nagahashi M., Kim E.Y., Harikumar K.B., Yamada A., Huang W.C., Hait N.C., Allegood J.C., Price M.M., Avni D. (2013). Sphingosine-1-phosphate links persistent STAT3 activation, chronic intestinal inflammation, and development of colitis-associated cancer. Cancer Cell.

[178] Yan Z., Zhan C., Wen Z., Feng L., Wang F., Liu Y., Yang X., Dong Q., Liu M., Lu W. (2011). LyP-1-conjugated doxorubicin-loaded liposomes suppress lymphatic metastasis by inhibiting lymph node metastases and destroying tumor lymphatics. Nanotechnology.

[179] Levet S., Ciais D., Merdzhanova G., Mallet C., Zimmers T.A., Lee S.J., Navarro F.P., Texier I., Feige J.J., Bailly S. (2013). Bone morphogenetic protein 9 (BMP9) controls lymphatic vessel maturation and valve formation. Blood.

[180] Ji R.C., Eshita Y. (2014). Rapamycin inhibition of CFA-induced lymphangiogenesis in PLN is independent of mast cells. Mol. Biol. Rep..

[181] Kim J.Y., Kim Y.J., Kim J.S., Ryu H.S., Lee H.K., Kang J.S., Kim H.M., Hong J.T., Kim Y., Han S.B. (2011). Adjuvant effect of a natural TLR4 ligand on dendritic cell-based cancer immunotherapy. Cancer Lett..

[182] Qiao J., Kottke T., Willmon C., Galivo F., Wongthida P., Diaz R.M., Thompson J., Ryno P., Barber G.N., Chester J. (2008). Purging metastases in lymphoid organs using a combination of antigen-nonspecific adoptive T cell therapy, oncolytic virotherapy and immunotherapy. Nat. Med..

[183] Kang T.H., Mao C.P., Lee S.Y., Chen A., Lee J.H., Kim T.W., Alvarez R.D., Roden R.B., Pardoll D., Hung C.F. (2013). Chemotherapy acts as an adjuvant to convert the tumor microenvironment into a highly permissive state for vaccination-induced antitumor immunity. Cancer Res..

[184] Tammela T., Saaristo A., Holopainen T., Lyytikkä J., Kotronen A., Pitkonen M., Abo-Ramadan U., Ylä-Herttuala S., Petrova T.V., Alitalo K. (2007). Therapeutic differentiation and maturation of lymphatic vessels after lymph node dissection and transplantation. Nat. Med..

[185] Christiansen A., Detmar M. (2011). Lymphangiogenesis and cancer. Genes Cancer.

[186] Bron S., Henry L., Faes-Van’t Hull E., Turrini R., Vanhecke D., Guex N., Ifticene-Treboux A., Marina Iancu E., Semilietof A., Rufer N. (2015). TIE-2-expressing monocytes are lymphangiogenic and associate specifically with lymphatics of human breast cancer. Oncoimmunology.

[187] Klein K.R., Caron K.M. (2015). Adrenomedullin in lymphangiogenesis: From development to disease. Cell. Mol. Life Sci..

[188] Kourtis I.C., Hirosue S., de Titta A., Kontos S., Stegmann T., Hubbell J.A., Swartz M.A. (2013). Peripherally administered nanoparticles target monocytic myeloid cells, secondary lymphoid organs and tumors in mice. PLoS ONE.

[189] Onishi Y., Eshita Y., Ji R.C., Onishi M., Kobayashi T., Mizuno M., Yoshida J., Kubota N. (2014). Anticancer efficacy of a supramolecular complex of a 2-diethylaminoethyl-dextran—MMA graft copolymer and paclitaxel used as an artificial enzyme. Beilstein J. Nanotechnol..

[190] Khan I.N., Al-Karim S., Bora R.S., Chaudhary A.G., Saini K.S. (2015). Cancer stem cells: A challenging paradigm for designing targeted drug therapies. Drug Discov. Today.

[191] Li S., Li Q. (2015). Cancer stem cells, lymphangiogenesis, and lymphatic metastasis. Cancer Lett..

[192] Clarke M.F., Dick J.E., Dirks P.B., Eaves C.J., Jamieson C.H., Jones D.L., Visvader J., Weissman I.L., Wahl G.M. (2006). Cancer stem cells—Perspectives on current status and future directions: AACR Workshop on cancer stem cells. Cancer Res..

[193] Karaman S., Detmar M. (2014). Mechanisms of lymphatic metastasis. J. Clin. Investig..

[194] Petitt M., Allison A., Shimoni T., Uchida T., Raimer S., Kelly B. (2009). Lymphatic invasion detected by D2-40/S-100 dual immunohistochemistry does not predict sentinel lymph node status in melanoma. J. Am. Acad. Dermatol..

